# 2-D Structure of the A Region of Xist RNA and Its Implication for PRC2 Association

**DOI:** 10.1371/journal.pbio.1000276

**Published:** 2010-01-05

**Authors:** Sylvain Maenner, Magali Blaud, Laetitia Fouillen, Anne Savoye, Virginie Marchand, Agnès Dubois, Sarah Sanglier-Cianférani, Alain Van Dorsselaer, Philippe Clerc, Philip Avner, Athanase Visvikis, Christiane Branlant

**Affiliations:** 1 AREMS, Nancy Université, UMR 7214 CNRS-UHP 1, Faculté des Sciences et Techniques, BP 70239, Vandoeuvre-lès-Nancy, France; 2 Laboratoire de Spectrométrie de Masse BioOrganique, Institut Pluridisciplinaire Hubert Curien, Département des Sciences Analytiques, Université de Strasbourg, CNRS UMR 7178, ECPM, Strasbourg, France; 3 Génétique Moléculaire Murine, CNRS2578, Institut Pasteur, Paris, France; Washington University School of Medicine, United States of America

## Abstract

In placental mammals, inactivation of one of the X chromosomes in female cells ensures sex chromosome dosage compensation. The 17 kb non-coding Xist RNA is crucial to this process and accumulates on the future inactive X chromosome. The most conserved Xist RNA region, the A region, contains eight or nine repeats separated by U-rich spacers. It is implicated in the recruitment of late inactivated X genes to the silencing compartment and likely in the recruitment of complex PRC2. Little is known about the structure of the A region and more generally about Xist RNA structure. Knowledge of its structure is restricted to an NMR study of a single A repeat element. Our study is the first experimental analysis of the structure of the entire A region in solution. By the use of chemical and enzymatic probes and FRET experiments, using oligonucleotides carrying fluorescent dyes, we resolved problems linked to sequence redundancies and established a 2-D structure for the A region that contains two long stem-loop structures each including four repeats. Interactions formed between repeats and between repeats and spacers stabilize these structures. Conservation of the spacer terminal sequences allows formation of such structures in all sequenced Xist RNAs. By combination of RNP affinity chromatography, immunoprecipitation assays, mass spectrometry, and Western blot analysis, we demonstrate that the A region can associate with components of the PRC2 complex in mouse ES cell nuclear extracts. Whilst a single four-repeat motif is able to associate with components of this complex, recruitment of Suz12 is clearly more efficient when the entire A region is present. Our data with their emphasis on the importance of inter-repeat pairing change fundamentally our conception of the 2-D structure of the A region of Xist RNA and support its possible implication in recruitment of the PRC2 complex.

## Abstract 2

Structural analyses provide new insights into the folding of the A region of the Xist RNA, which plays a crucial role in X chromosome inactivation, and its mechanism of protein recruitment.

## Author Summary

In placental mammal females, Xist RNA is crucial for inactivation of one of the two X chromosomes in order to maintain proper X chromosome dosage. It is known that the conserved A region of Xist RNA, which contains eight or nine repeated elements, plays an essential role in this process, however, little is known about its structure and mechanism of action. By using chemical and enzymatic probes, as well as FRET experiments, we performed the first experimental analysis of the solution structure of the entire Xist A region. Both mouse and human A regions were found to form two long stem-loop structures each containing four repeats. In contrast to previous predictions, interactions take place both between repeats and between repeats and spacers. Affinity-purification of RNA-protein complexes formed by incubation of RNA in mouse ES cell nuclear extract, followed by mass spectrometry and antibody-based analyses of their protein contents, showed that the isolated 4-repeat structures from the A region can recruit components of the PRC2 complex that is needed for X chromosome inactivation. However, association of one component of this complex, Suz12, was more efficient when the entire A region was used.

## Introduction

In mammals, the transcriptional silencing of one of the two X chromosomes in female cells (X chromosome inactivation, XCI) ensures sex chromosome dosage compensation [1]. Once acquired early in development, the inactivated state is faithfully inherited through successive cell divisions. XCI initiation is associated with increased Xist RNA transcription. Whilst first retained near its transcription site, Xist RNA then spreads along the entire X chromosome from which it has been transcribed [2]–[5] whilst, a series of epigenetic marks, which include the repressive histone modifications H3K27me3, H3K9me3, are recruited to the presumptive inactive X chromosome. Xist RNA is a long non-coding RNA (17 kb in length in the mouse), which is capped, spliced, and polyadenylated. Little is known about its structure and mechanism of action.

The Xist gene has a complex origin. It includes degenerated pieces of an ancient protein gene *Lnx3* as well as genomic repeat elements derived at least in part from transposon integration events [6],[7]. The most conserved Xist RNA regions correspond to repeat elements (denoted A to E in mouse [8]), which are organized as tandem arrays. The A region (positions 292 to 713 in mouse, accession no. gi|37704378|ref|NR_001463.2| [2], and 350 to 770 in human, accession no. gi|340393|gb|M97168.1| [5]) is the most highly conserved of the repeat regions and is critical for initiation of XCI. The observation that female mouse embryos carrying a mutated Xist^ΔA^ gene inherited from males are selectively lost during embryogenesis underlines the importance of this element [9]. Recent data have shown that an early event in silencing is the formation of a Xist RNA compartment and that the A region whilst not necessary for formation of this compartment is needed for relocation of X linked genes into this territory [10]. Over-expression of a Xist^ΔA^ RNA in transgenic mouse ES cells indicates that the A region whilst not necessary for Xist coating is implicated in the recruitment of the PRC2 complex [11]–[16]. The PRC2 complex contains the Suz12, Eed, Ezh2, and Rbap46–48 proteins [17],[18]. Eed and Suz12 have been proposed to bind nucleic acids [19],[20], whereas Rbap46–48 may interact with nucleosome protein components [17]. Lysine 27 tri-methylation of histone H3 is catalysed by Ezh2 [12],[14] and both Eed and Suz12 are required for this activity [20]. Recently a short 1,600 nucleotide-long RNA which contains the A region at its 5′ extremity was suggested to be expressed early in XCI initiation and to bind the PRC2 complex [21].

Since the function of Xist RNA is expected to depend on its 2-D structure, studies aimed at establishing the 2-D structure of the Xist A region have considerable interest. Based on nucleotide sequence of the A region and computer prediction, Wutz and colleagues have proposed that each repeat forms two short stem-loop structures [11]. Recent NMR analysis confirmed that one of these stem-loop structures can be formed in vitro by an RNA molecule bearing a single copy of the mouse repeat A sequences [22]. In Xist RNA, the repeat sequences are, however, separated by long spacer regions (21 to 48 nt long for mouse). Since current models fail to take account of this sequence complexity, an experimental analysis of the entire A region was thought likely to provide valuable information on the structure of the A region. As conventional probing experiments are, however, hindered by the presence of the repeated sequences and long U tracks, we applied a combined approach exploiting both chemical and enzymatic probing of RNA structure in solution and FRET experiments using fluorescent oligonucleotide probes complementary to different parts of the A region.

Using this dual approach, we could show that repeats in the A region interact with each other to form long irregular stem-loop structures. Such inter-repeat interactions appear to be required for the binding of the various components of the PRC2 complex. We identified the minimal number of repeats necessary for such binding. The implications of our results within the wider context of X-inactivation and of the XCI mechanism(s) underlying silencing are discussed.

## Results

### Probing of Mouse and Human A Region 2-D Structures

**Figure 1:**
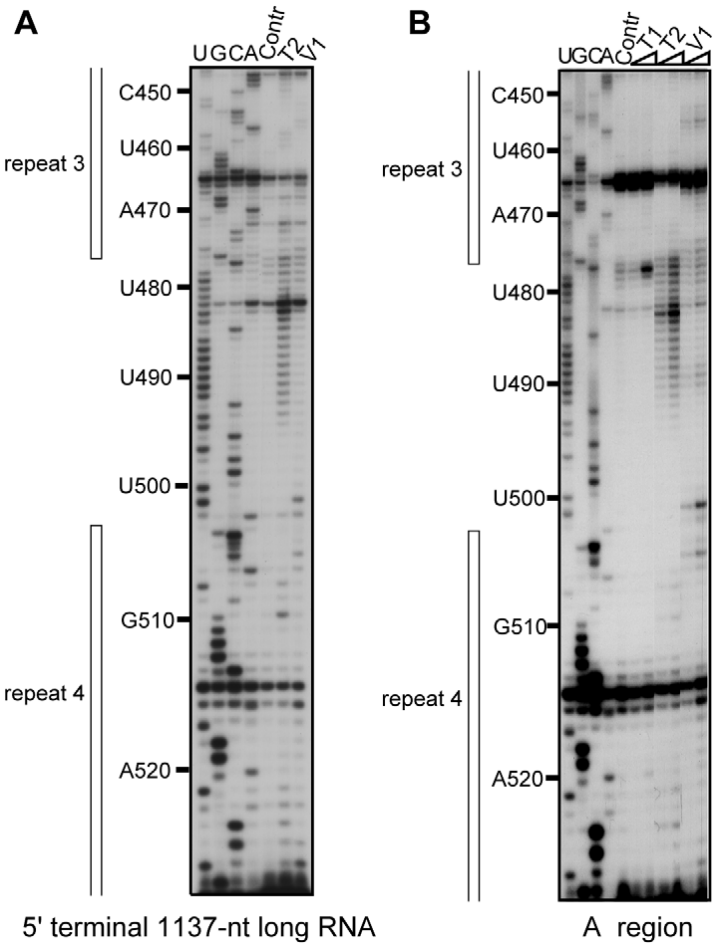
Probing of mouse Xist A region RNA structure alone and inside 5′-terminal region. The two RNAs (A. for 5′ terminal 1137-nt long RNA, B. for A region) were in vitro transcribed and renatured as described in Material and Methods, before being subjected to limited digestion with T1, T2, or V1 RNases under the conditions described in Materials and Methods. Extension analyses were performed using oligonucleotide 3866 (Table S1) as the primer. The resulting cDNAs were fractionated by electrophoresis on 7% denaturing polyacrylamide gel. Lanes U, G, C, and A correspond to the sequencing ladder obtained with the same primer. Lanes marked by Contr corresponds to primer extension analysis of undigested RNA transcripts. Nucleotide numbering on the left-hand side of the autoradiogram takes the first residue of mouse Xist RNA as residue 1. The sequences corresponding to repeats 3 and 4 are indicated by vertical bars on the right-hand side of the autoradiograms.

We analysed in parallel both the entire mouse and human A regions, as the sequence divergence of the inter-repeat linking sequence between mouse and human was expected to provide insight into how the spacer regions might influence the A repeat structure. The specific primers for the two A regions are listed in Table S1. To test if the A region interacts with neighbouring Xist RNA sequences, an RNA that contained only the mouse A region (positions 277 to 760 in mouse Xist RNA) and a larger RNA including the sequence extending from positions 1 to 1137 were studied in parallel by limited enzymatic digestion. Very similar digestion profiles (Figure 1A and B) were obtained for the two RNAs when digestions were performed on the T7 RNA transcripts after folding under the conditions outlined in Materials and Methods. We conclude that the A region probably folds on itself without major interaction with other upstream and downstream Xist sequences. Hence, our subsequent analyses of the 2-D structure of the Xist A region were carried out in the absence of flanking sequences (positions 227 to 760 and 330 to 796 for the mouse and human A regions, respectively). Each enzymatic digestion and chemical modification assay was carried out in duplicate using different transcript preparations and each extension analysis repeated 2 or 3 times for each primer. Representative examples of the primer extension analyses are provided in Figure 1 and Figure S1A–S1E.

### M-Fold Assisted Modelling of the Mouse A Region 2-D Structure

**Figure 2:**
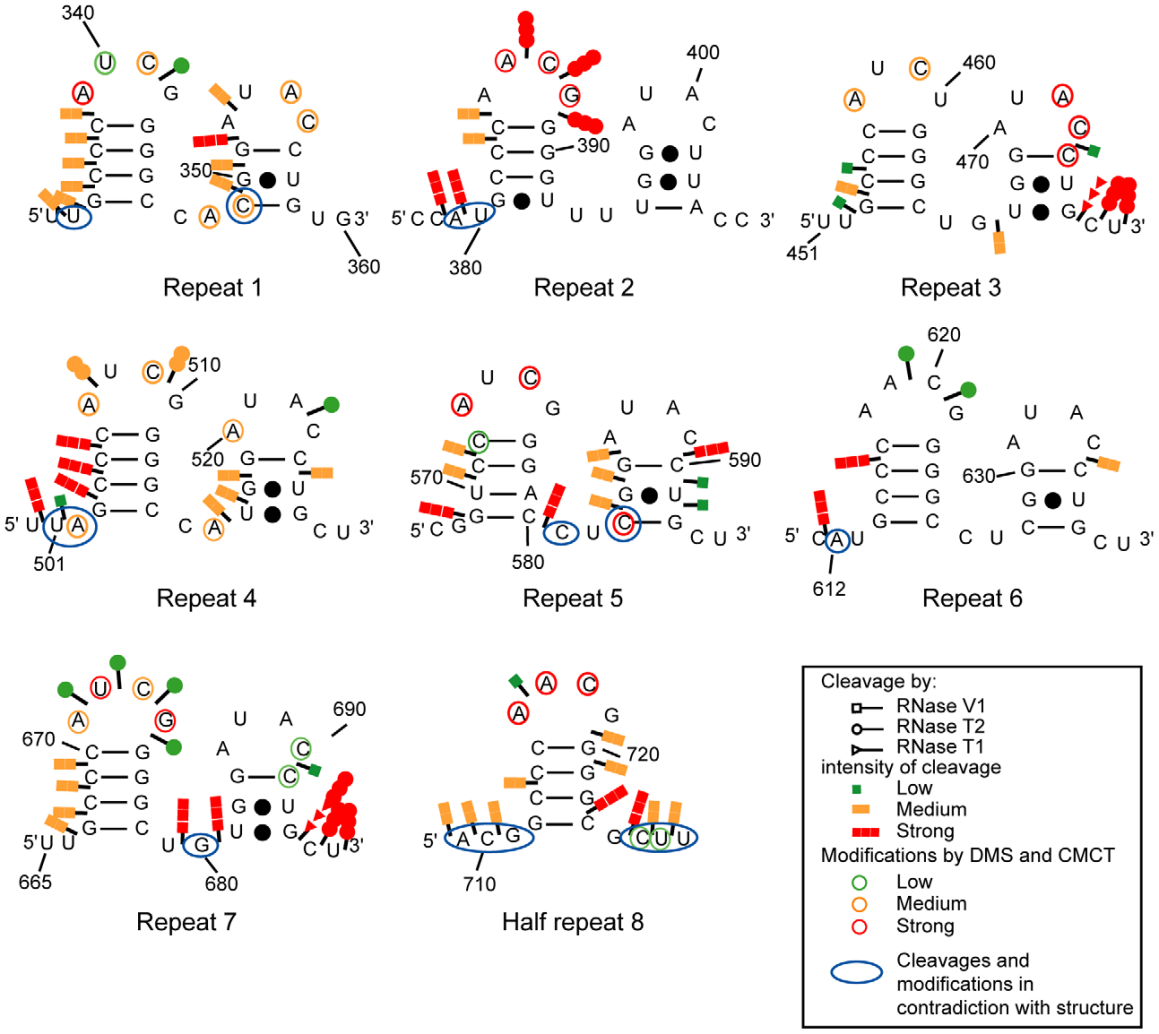
Representation of experimental data on the previously proposed 2-D structure of the Xist A region. Each of the seven repeats as well as the eighth half repeat from the mouse A region was folded according to the previously predicted two stem-loop structure [11]. T1, T2, and V1 RNase cleavages were represented by arrows surmounted by circles, triangles, and squares, respectively. Nucleotides modified by DMS or CMCT are circled. Colours of circles and arrows indicate the yields of modifications and cleavages—red, yellow, and green for strong, medium, and low modification or cleavage, respectively. The V1 RNase cleavages and chemical modifications that cannot be explained by the two stem-loop structure models are encircled by blue lanes.

**Figure 3:**
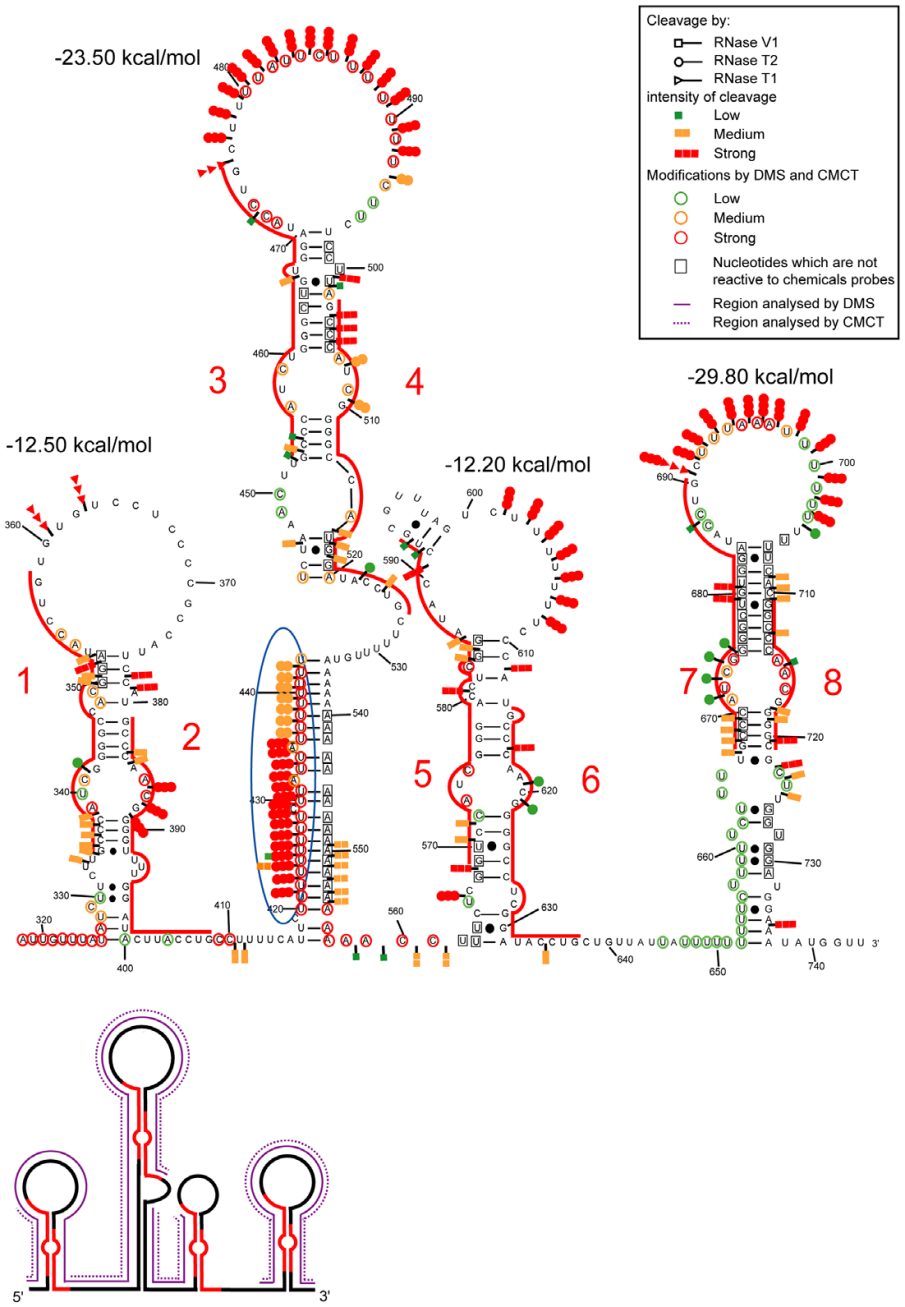
Representation of experimental data on the possible 2-D structure 1 of the Xist A region. In model 1, the various stem-loop structures all involve two successive repeats. The repeats are indicated by red lines and are numbered from 1 to 8. Representations of chemical and enzymatic data are as in Figure 2. In each of the three panels, segments in which DMS and/or CMCT modifications were identified are indicated on a schematic drawing of the 2-D structure (full and dot lines for DMS and CMCT, respectively). In segments analyzed by the two chemical reagents, unmodified nucleotides are squared. However, one should take into consideration the fact that G residues are poorly modified by CMCT in the mild conditions that we used. The free energies of each stem-loop structures at 0°C and in 3 M NaCl were calculated with the M-fold software.

**Figure 4 pbio-1000276-g004:**
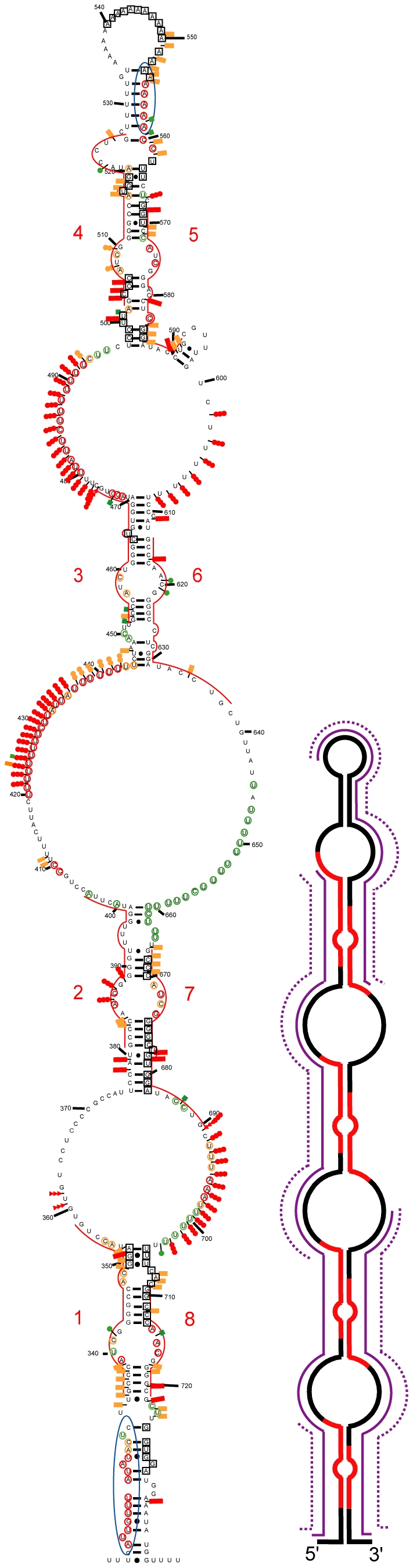
Representation of experimental data on the possible 2-D structure 2 of the Xist A region. In model 2, repeats in the 5′ half of the A region interact with repeats in the 3′ half of this region. Representation of enzymatic cleavages and chemical modifications are as in Figures 2 and 3.

**Figure 5:**
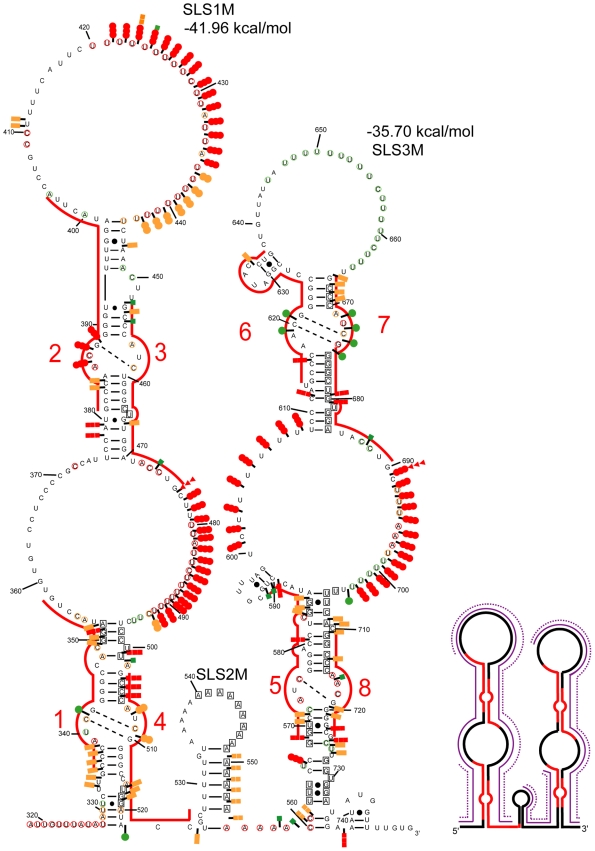
Representation of experimental data on the possible 2-D structure 3 of the Xist A region. In model 3, two stem-loop structures containing four repeats are separated by a small stem-loop corresponding to poly A and poly U sequences. Representation of enzymatic cleavages and chemical modifications are as in Figures 2 and 3.

The structure proposed by Wutz and colleagues, in which each of the repeats fold into a double stem-loop structure, could not explain the numerous V1 RNase cleavages that we observed with both the mouse and human A regions (Figure 2) [11]. We explored the possibility that each repeat folds into a unique longer stem-loop structure. Such folding was similarly unable to explain V1 RNase cleavages (Figure S2). We conclude that the 2-D structure may involve interactions between repeats and spacers and inter-repeat interactions. There is, however, a multitude of potential ways for duplex formation between repeats (Figures 3–5, models 1–3). Our design of the putative structure was orientated by the detection of six successive strong V1 RNase cleavages in the central poly A sequence (positions 550 to 555), suggesting the involvement of this segment in a helical structure. The strong modification by DMS of a sequence immediately downstream (positions 555 to 561) was an indication for a single-stranded state. One possible explanation for these data was the formation of a central stem-loop structure called SLS2M with a U track on one strand and an A track on the other strand (Figure 5). Formation of this central stem-loop structure was subsequently imposed as a constraint when exploring the possible folding of the mouse A region. This excluded structures in which two successive repeats would interact with each other (Model 1, Figure 3), since in this case, the entire poly A region would interact with the poly U track located upstream of repeat 3, which was not in agreement with the probing data (Figure 3). Another possible structure involved formation of an interaction between the 5′ and 3′ halves of the A region. This would generate a very long irregular stem-loop structure with an A rich terminal loop (Model 2, Figure 4). An alternative structure involved the folding on themselves of the 5′ and 3′ parts of the A region, with SLS2M in between (Model 3, Figure 5). Several other alternative pairings of the repeats were also explored—none fitted the chemical and enzymatic data perfectly.

The notion of independent folding of the 5′ part of the A region (positions 318 to 521 in mouse RNA) was supported by M-fold analysis of this segment, which identified a highly stable long irregular stem-loop structure, SLS1M (ΔG = −41.96 kcal/mol at 0°C in 3 M NaCl), in which repeat 1 interacts with repeat 4 and repeat 2 interacts with repeat 3. It is the most thermodynamically stable structure proposed for this 5′ segment and was predicted irrespective of whether the experimental data were introduced as a constraint in the M-fold search. In SLS1M, each repeat interacts both with another repeat and with a spacer segment, increasing the stability of the overall structure. Similarly, M-fold analysis of the 3′ part of the mouse A region suggested that repeat 5 interacts with repeat 8 and a spacer region and repeat 6 with repeat 7 and a spacer region. The resulting SLS3M structure was predicted as the most favourable structure by M-fold when the experimental data were incorporated as a constraint. The overall predicted three stem-loop structure (Model 3) has a low calculated free energy (−77.76 kcal/mol) and has a better fit to the experimental data than Models 1 and 2 (Figures 3 and 4), suggesting that, in solution, Model 3 is the most likely structure among the numerous possibilities.

### The Possibility to Form Structure 3 Is Phylogenetically Conserved

**Figure 6:**
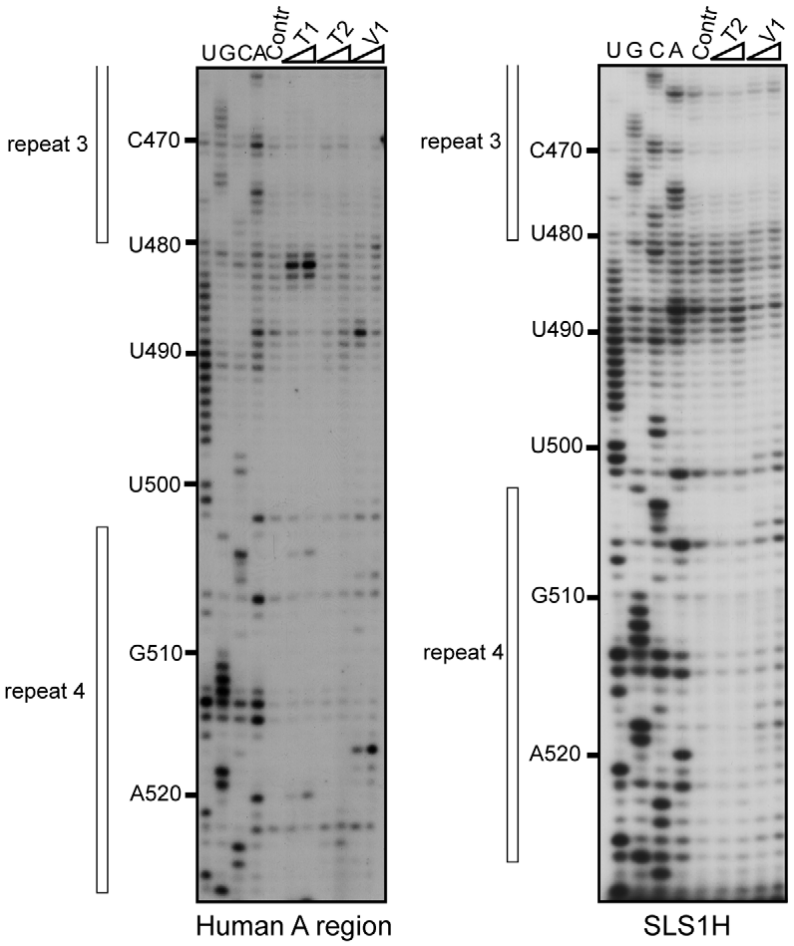
Probing of the 2-D structures of the entire and 5′ half of human A region. Legend as in Figure 1, except that the transcripts correspond to the entire human A repeat region (human A RNA) and the 5′ half of the human A region (SLS1H RNA), respectively.

**Figure 7:**
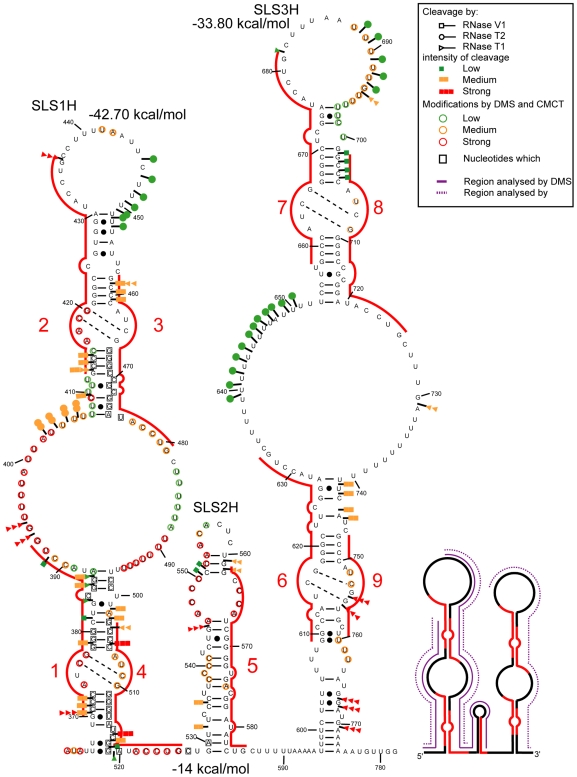
Representation of experimental data on possible structure 3 of human Xist A region. Repeat 5 is located in a short central stem-loop structure flanked by two larger stem-loop structures that each contain four repeats. Representation of the enzymatic cleavages and chemical modifications are as in Figures 2 and 3. Indication of segments in which DMS and/or CMCT modifications were identified is represented as in Figure 3. The free energies of the stem-loop structures were calculated with the M-fold software.

If a structure has biological relevance, it is generally conserved throughout evolution. Therefore, we tested whether the most favourable structures identified for the mouse A region were relevant to the human A region in solution. The sequence of the human A region differs from that of mouse by the presence of an additional repeat 5 and the absence of a long central polyA region. Experimental data (Figures 6 and 7 and Figure S3A–E) suggested that the central repeat 5 forms a central stem-loop structure (SLS2H). Based on this, structures similar to mouse Models 2 and 3 could be proposed for the human A region which involve either a long irregular stem-loop structure including all the repeats (Model 2) or a three stem-loop structure (Model 3) with repeats 1 to 4 forming a first stem-loop structure (SLS1H), repeat 5 folded alone in a second stem-loop (SLS2H) and repeats 6 to 9 involved in a third stem-loop structure (SLS3H). As for the mouse A region: (i) A 2-D structure in which each repeat interacts with its immediate downstream repeat (repeats 1, 3, 6, and 8 with repeats 2, 4, 7, and 9, respectively, Model 1) was not supported by the probing data (segment 688 to 696) (Figure S4); (ii) M-fold analysis of the 5′ portion of the human A region (positions 370 to 530) either with or without the experimental data as a constraint identified SLS1H as the most stable structure (ΔG = −42.70 kcal/mol) (Figure 7); (iii) SLS3H was retained as the most stable structure for the 3′ part of the A region, when the experimental data were added as a constraint to an M-fold search; and (iv) the 3 stem-loop structure corresponding to Model 3 (ΔG = −86.6 kcal/mol) had the best fit with probing data compared to the other 2-D models. Further support for Model 3 was provided by our observation of identical patterns of enzymatic cleavage for the entire human A region and for the isolated SLS1H portion (Figure 6).

The maintenance of interactions between both spacers and repeats during mammalian evolution of the A region implies that the nucleotide sequences involved in these interactions were either conserved or subjected to compensatory base changes. This was confirmed by the alignment of the mouse, human, orangutan, baboon, lemur, dog, rabbit, cow, and elephant A region sequences (Figure S5). Nucleotide sequence conservation extends out beyond the repeats themselves for the majority of the repeats, allowing formation of the SLS1 and SLS3 structures in all sequenced Xist RNAs (Figure S6).

### FRET Experiments Bring Additional Data in Favour of Model 3

**Figure 8:**
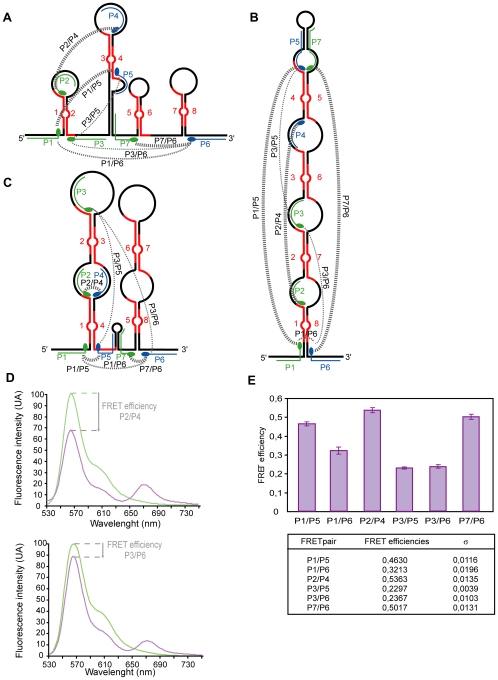
Steady-state fluorescence studies provide additional support to the A region Model 3. In (A, B, and C), the binding sites of oligonucleotides P1 to P6 used in the FRET experiments are shown for the three 2-D structures of the A region corresponding to Models 1, 2, and 3. The identity of the chromophore present in each oligonucleotide (donor Cy3 or acceptor Cy5) is indicated in green and blue, respectively. Cy3- and Cy5-labeled oligonucleotides were purchased from Eurogentec. As illustrated in (D), the emission fluorescence spectra from 530 to 745 nm of the donor oligonucleotide bound alone to the RNA (green curve) were collected, as well as the emission spectra obtained in the presence of the donor and acceptor oligonucleotides (violin curve). No energy transfer between Cy3- and Cy5-labeled oligonucleotides was detected in solution. The FRET efficiency for each pair of oligonucleotides was defined as the decrease in fluorescence of the donor at 564 nm in the presence of the acceptor. Two representative examples of FRET assays (oligonucleotide pairs P2/P4 and P3/P6) are shown in (D). The FRET efficiencies measured for the six pairs of oligonucleotides are provided in (E) (mean values of three independent experiments). Standard deviations (σ) are shown. The relative efficiencies of the FRET obtained for each oligonucleotide pairs are schematically represented in (A, B, and C) by lines joining the oligonucleotides. The thickness of the lines reflects the efficiency of the FRET effect.

Three oligonucleotide pairs (P1–P5, P2–P4, and P6–P7) were retained in order to test Model 3 by FRET experiments (Figure 8). This Model predicts that the P1–P5 and P2–P4 pairs of oligonucleotides interact with the single-stranded segments which border the helix formed by repeats 1 and 4, whilst the P6–P7 pair of oligonucleotides should interact with the single-stranded segments bordering the helix formed by repeats 5 and 8. A marked FRET effect would therefore be expected for these three oligonucleotide pairs if the A region was folded as in Model 3. The distance between the fluorophores of these three pairs of oligonucleotides would, on the other hand, be expected to be much larger if region A was folded as in structures 1 or 2. Whilst tertiary structural interactions might decrease the distances, a lower level of FRET would still be expected to be observed for the three pairs of oligonucleotides if the A region was folded according to structures 1 or 2 (Figure 8). The P1 and P6 oligonucleotides bind to two single-stranded segments which flank the helix formed by repeats 1 and 8 in structure 2. A strong FRET effect for P1 and P6 would therefore be expected if the A region was folded according to structure 2. Upon binding to the A region, oligonucleotide P7 partially disrupts the base-pair interactions formed by the central poly A stretch. However, as similar levels of destabilization are expected for the three possible structures, binding of this oligonucleotide was not expected to favour one structure more than the two other ones. The same is true for oligonucleotide P5 that binds to the partner U stretch of the poly A sequence. To monitor the level of FRET obtained for oligonucleotides bordering a helix, we used the short R1–2 transcript containing repeats 1 and 2 and their bordering sequences, which adopt a single unique 2-D structure and the P1–P3′ oligonucleotide pair (Figure S7). Other controls exploited the P3–P6 and P3–P5 pairs, which were not expected to be in close proximity in any of the three proposed structures (Figure 8). The oligonucleotide pairs used are shown in Figure 8, along with examples of typical fluorescence intensity spectra recorded in FRET experiments for the P2–P4 and P3–P6 pairs (Figure 8D). High FRET signals in the range of 50% were obtained for the P1/P5, P2/P4, and P7/P6 oligonucleotide pairs, whilst lower FRET signals were observed for the P1–P6 (35%) and especially the P3–P6 and P3–P5 oligonucleotide pairs (25% and 22%, respectively). This is compatible with a large part of the molecules being folded in solution into structure 3. Folding of a large number of molecules into structure 2 would have led to a strong FRET signal for the P1–P6 pair and lower signals for the five other pairs, which was not observed. The strong FRET effects obtained for the P1/P5, P2/P4, and P7/P6 oligonucleotide pairs argues strongly against folding according to structure 1. Based on our FRET data, we conclude that folding predominantly occurs according to Model 3.

### Recruitment of the PRC2 Complex by the A Region

Previous studies have shown that the PRC2 complex interacts with the Xi [12],[14],[15],[23] and the A region has been proposed to recruit the PRC2 complex through the Ezh2 subunit, which would act as an RNA-binding subunit [21]. We wished to explore further the binding of the PRC2 complex to the A region in the light of our structural data. In particular, we were interested in determining how many A region repeats were required to bind the individual Eed, Ezh2, RbAp46, RbAp48, and Suz12 components of the PRC2 complex.

**Figure 9:**
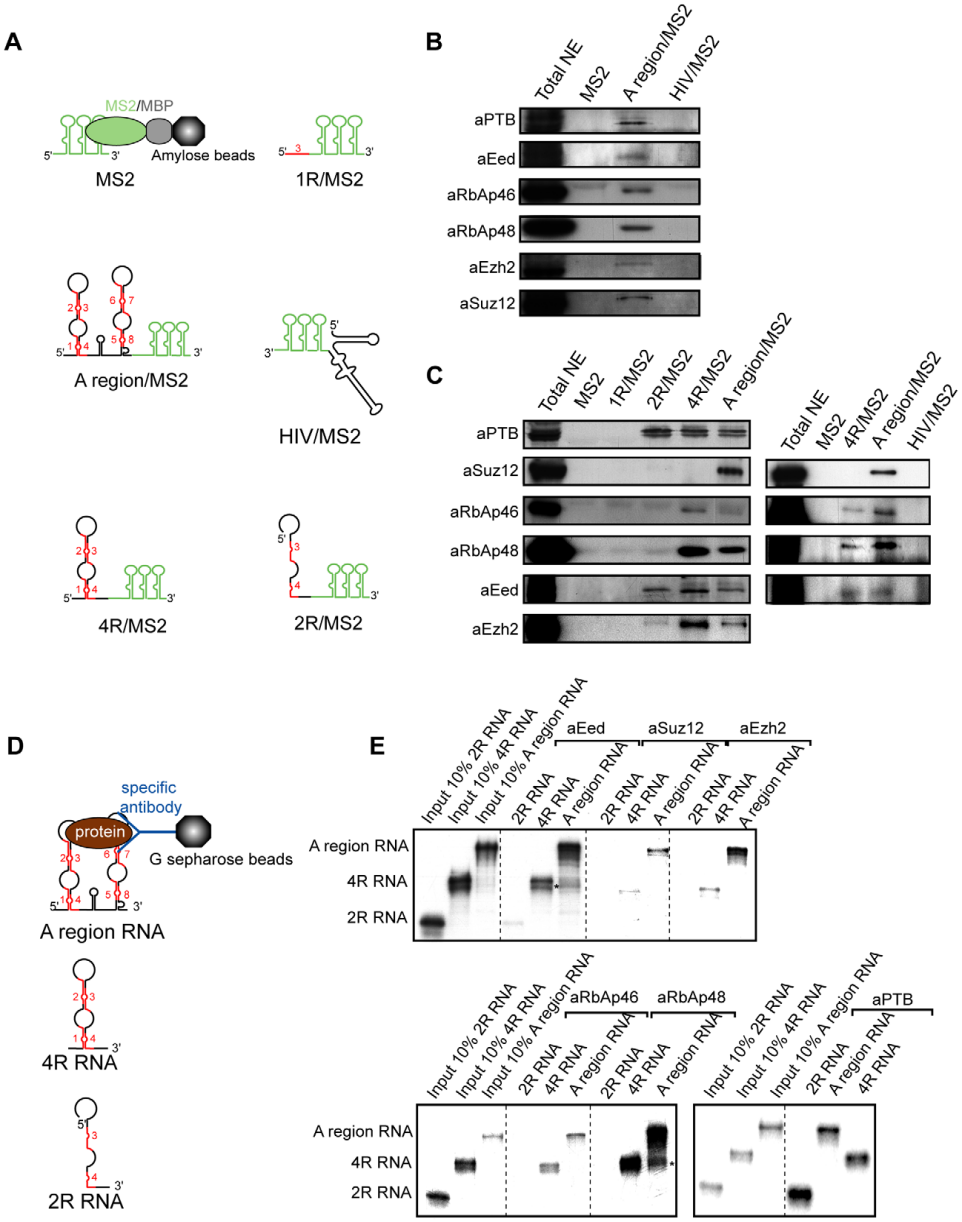
The PRC2 complex assembles on fragments of the A region containing at least four repeats. (A) Representation of the fusion RNAs used for formation of RNP complexes with ES cell nuclear extracts. Retention of RNA containing three MS2 coat protein binding sites (MS2) on amylose beads was mediated by the MS2/MBP fusion protein [31]. Analysis of the protein content of the RNP complexes formed on the A region/MS2 RNA was achieved by mass spectrometry (Figure S8). (B and C) Western blot assays using antibodies specific for the PTB, Suz12, RbAp46, RbAp48, Eed, and Ezh2 protein were used to evaluate the relative amounts of these proteins in the purified complexes formed with the various RNAs shown in (A). Antibodies were purchased from Santa Cruz (Sc), Abcam (ab), and Calbiochem (cb): anti-Ezh2 (anti-ENX-1 H-80, sc-25383), anti-RbAp46 (ab3535), anti-RbAp48 (ab488), anti-Eed (ab4469), anti-Suz12 (ab12073), and anti-PTB (cb NA63). (D and E) Test of the association of radiolabelled fragments of the A region with components of the PRC2 complex present in nuclear extracts of ES cells. The three RNAs tested are represented in (D). RNAs bound to the G sepharose beads were fractionated by electrophoresis on 7% denaturing gels. Autoradiograms of the gels are shown in (E). Input corresponds to 10% of the material incubated with the beads.

We initiated a proteomic approach based on affinity chromatography purification of complexes formed upon incubation of in vitro transcribed Xist A region RNAs with nuclear extracts, followed by protein identification by mass spectrometry and Western blot analysis. Mouse ES cells are a widely exploited model for the study of XCI initiation, and we reasoned that as Xist RNA acts as an initiator of XCI, proteins which have to interact with this RNA to ensure early Xist functions should already be present in the nuclear extract of ES female mouse cells prior to differentiation. We used a control RNA containing only the three MS2 protein binding sites and tested four RNAs containing different segments of the mouse Xist A region flanked by the three MS2 binding sites at their 3′ end (Figure 9A). These RNAs denoted as 1R/MS2, 2R/MS2, 4R/MS2, Aregion/MS2, and HIV/MS2 contained, respectively, repeat 1 without any neighbouring sequence, repeats 3 and 4 and their bordering spacers (positions 401 to 552 in mouse Xist RNA), the SLS1M stem-loop structure, the entire A region, and a fragment of HIV-1 RNA (positions 5338 to 5514 in the BRU RNA) used as a negative control.

In order to get an idea of the proteins capable of associating with the entire A region, the proteins bound to purified complexes formed on the Aregion/MS2 RNA were analysed by mass spectrometry. Among numerous proteins detected were protein PTB and components of the PRC2 complex (Ezh2, RbAp46, RbAp48, and Suz12) (Figure S8). We then evaluated by Western blot experiments the relative amounts of each of the PRC2 components in RNPs formed by the various RNAs tested. Whilst Eed, Ezh2, and PTB were detected in complexes formed on RNAs containing two or more repeats, binding of RbAp46 and RbAp48 was detected only when using RNAs with at least four repeats and Suz12 when the entire A region was used (Figure 9C). The control HIV-1 RNA bound none of these proteins (Figure 9B and 9C).

To further explore these data, we performed a series of experiments in which fragments of the A region were transcribed in vitro as radio-labelled RNA without MS2 fusion and these RNAs were incubated with mouse ES nuclear extracts. Three distinct RNAs (the complete A region, 4R, and 2R RNAs; Figure 9D and 9E) were used for these experiments.

In confirmation of the possible interaction of Eed with an RNA containing only two repeats, trace amounts of 2R RNA were retained on the beads when an anti-Eed antibody was used. Only complexes containing the 4R RNA or the entire A region were retained when anti-Suz12, anti-Ezh2, anti-RbAp46, and anti-RbAp48 antibodies were used. These observations confirmed the importance of the corresponding regions for association of these proteins. Clearly, however, higher amounts of the entire A region compared to 4R RNA were bound when the anti-Suz12 and Ezh2 antibodies were used.

We conclude that whilst some segments of the A region allow the binding of particular PRC2 components, the entire A region is required for efficient association of the entire complex.

## Discussion

The A region of Xist RNA plays an essential role in the X inactivation process. Here, we show that in vitro the repeated elements in the A region of both mouse and human Xist RNAs interact together and with the intervening spacer regions. This leads to the formation of peculiar long irregular stem-loop structures containing four repeats and long U rich terminal and internal loops. Our proteomic analysis suggests that these four-repeat structures may correspond to functional modules initiating the assembly of the PRC2 complex.

### A New Conception of the 2-D Structure of the A Region

Until now, both computer [11] and experimental analyses [22] of the possible 2-D structure of the A region of Xist RNA have privileged the individual repeat as the unit of folding. However, the presence of long intervening spacer sequences between the repeats suggests that these spacer sequences may participate in 2-D structure formation, and points to the potential inadequacy of previous models. Our detailed chemical and enzymatic probing of the A region structure in solution involving the design of specific primers for reverse transcriptase extension analysis enabled us to identify for the first time the double-stranded and single-stranded segments making up the A region structure in solution. The data obtained clearly demonstrate that the repeats do not fold on themselves but rather fold one with the other.

Chemical and enzymatic probing of an RNA structure in solution often allows the building of a unique 2-D structure model in agreement with the experimental data. Studies on the A region were, however, complicated by the high degree of sequence redundancy. Use of a recently proposed biophysical approach, based on FRET assays [24], helped overcome these difficulties by providing information on the relative distances between the sequences flanking the various repeats. To our knowledge, up to now, this approach has only been used to confirm the 2-D structure model of telomerase RNA [24]. This method, which involves the utilization of oligonucleotides carrying donor and acceptor fluorescent dyes complementary to single-stranded segments in the studied RNA, proved particularly well suited to the study of the A region, since our probing data identified several long single-stranded segments which were able to bind the oligonucleotide probes. Among the possible 2-D structures for the A region, only one, structure 3, showed perfect agreement with the FRET data. Structure 3, which contains two long irregular stem-loop structures, each involving four repeats (four-repeat structure), also shows the best agreement with the chemical and enzymatic data. The two long stem-loop structures are separated by a short stem-loop structure, corresponding to a divergent region between mouse and human Xist RNAs. One repeat in this segment is common to all the sequenced Xist RNAs (Figure S5), except mouse RNA. In the latter, it is replaced by a poly A sequence forming a short stem loop with a poly U sequence. Interestingly, nucleotide sequence conservation in the A region extends to the spacer extremities, which contribute to the possibility of forming the four-repeat structures (Figure S5).

Although the presence of large internal and terminal loops decreases the stability of stem-loop structures, the predicted free energies of the two four-repeat structures in both mouse and human RNAs have strongly negative values (between −33 and −45 kcal/mol), explaining why they are proposed by the M-fold software. In addition, these four-repeat structures may be stabilized *in cellulo* by RNA-protein interactions. Interestingly, protein PTB, which contains 4 RNA recognition motifs (RRMs), which are each able to interact with UCUU(C), UUCUCU, or CUCUCU sequences, showed high affinity for the A region in nuclear extract binding experiments (Figure 9) [25]. As UCUU motifs are present on each side of the large internal loops in the four-repeat structures and in the terminal loop, one might imagine that interactions of the RRMs of a single PTB molecule with these various segments may stabilize the four-repeat structure as suggested by previously proposed models for protein PTB-RNA interaction [26].

In spite that model 3 has the best fit with all the data compared to other models, this does not exclude the possibility of some local dynamic in small areas of the SLS1 and SLS3 structures. More precisely, the instability of a few base-pair interactions can explain the presence of both V1 RNase cleavages and chemical modifications in a limited number of very small segments of the A repeat region.

### Possible Functional Implication of the Four-Repeat Structure

Our adaptation of the affinity purification chromatography, originally developed for purifying spliceosome complexes [27], to complexes formed upon incubation of different fragments of the A region with nuclear extracts prepared from undifferentiated mouse ES cells, coupled with mass spectrometry and Western blot analyses, was powerful. Together with immunoselection assays performed on assembled RNP complexes, it revealed the capability of four components of the PRC2 complex to associate with an RNA corresponding to one of the four-repeat structures formed by the A region. However, our observation that the entire A region is needed for efficient association of the Suz12 protein suggests a putative additional functional role for the entire A region in either binding Suz12 or in stabilising the binding of Suz12 to the four-repeat structure. This is too early to give a convincing molecular explanation of this observation. Further experiments are needed to understand why Suz12 displays different association properties compared to other members of the PRC2 complex.

Whilst UV cross-linking of the RNP complexes formed with ES nuclear extract using the entire A region has confirmed the direct binding of PTB to this RNA region, direct binding of components of the PRC2 complex was not detected (unpublished data). Neither Ezh2 nor Eed, which were previously proposed to be recruited by Xist in an A region dependent manner [21], were cross-linked in significant amounts, suggesting that their association with the A region is mediated via association with other nuclear proteins. Therefore, the peculiar SLS1 and SLS3 structures in the A region may be needed to recruit nuclear proteins which have an affinity for components of the PRC2 complex or to reinforce the RNA affinity for these components. Mass spectrometry analysis of RNP complexes formed with the entire A region showed that, in addition to components of the PRC2 complex, a large number of other nuclear proteins can associate with this RNA region. In further studies, it will be important to identify which of these proteins are required for PRC2 association and which ones bind directly to the A region structure.

Our finding that Suz12 requires the entire A region, or more simply more than four repeats for efficient association with the RNA, is in good agreement with the observation of Wutz and colleagues (2002) that the presence of at least 5.5 repeats is needed to initiate inactivation [11]. Additional support for the functional significance of the four-repeat model comes from a reworking of data obtained by Wutz and colleagues, who tested the effect of a series of mutations within the A region on XCI initiation [11]. Our structural studies show that all the variants (XR, XSR, XCR) classed by Wutz et al. as active are able to form the four-repeat structure, whereas the two inactive variants (XS1 and XNX) cannot (Figure S9).

Although several data argue in favour of a major role of the four-repeat structure in A repeat activity, we cannot exclude a possible role of alternative structures, for instance in modulating A repeat activity.

### Possible Implication of PRC2 and Other Nuclear Protein Association with the A Region

Although it is clear that the A region is essential for the X inactivation process, the precise role and mechanisms involved in the action of the A region remain unclear. Its deletion was shown to block silencing but not the coating of the X chromosome by Xist [11], an observation in agreement with a possible role of the A region in PRC2 recruitment. PRC2 is needed for apposition of some, but not all, of the epigenetic marks which are specific features of silenced chromatin in general and the inactive X in particular (methylation of histone H3 at position 27) [20]. The association of PRC2 with long ncRNAs before transfer of the PRC2 complex to chromatin may be a general mechanism for chromatin silencing processes that depend on long ncRNAs. Both the HOTAIR and Kcnq1ot1 long ncRNAs, which are involved in gene silencing, were recently found to bind the PRC2 complex [19],[28].

Recruitment of PRC2 is a relatively early event in X inactivation [14] in agreement with a possible early association of this complex with Xist RNA prior to extensive Xist coating of chromatin. One could imagine that PRC2 is associated with the chromatin upon Xist coating through its interaction with proteins bound to Xist RNA. Alternatively coating of the Xist RNP may facilitate PRC2 transfer to chromatin by interaction of some of the RNP components with proteins of the chromatin structure. Lee and colleagues recently reported the existence of the 1600 nucleotide long RepA RNA carrying the A region at its 5′ extremity, which may be expressed prior to expression of the entire Xist RNA and has been reported to recruit the PRC2 complex in a very early step of XCI [21]. Independent confirmation of these findings will be of major importance to the field.

Screening of the numerous other proteins that we have found to be capable of association with the entire A region by mass spectrometry for their eventual specific involvement in the recruitment of genes to the X inactivation domain [10] or other early events characterising the onset of X initiation and silencing will be of potential major importance to our understanding of X inactivation.

## Materials and Methods

### RNA Preparation

DNA fragments coding for the entire A regions of mouse and human Xist RNAs and their subfragments were PCR amplified using mouse or HeLa cell genomic DNA, and cloned into plasmid pUC18 under the control of a T7 promoter. RNAs were generated by run-off transcription with T7 RNA polymerase as previously described [29]. DNA templates were digested with RNAse-free DNAse I and RNA transcripts were purified on denaturing 3% to 8% polyacrylamide gels.

### Enzymatic and Chemical Probing of RNA Secondary Structure

RNA 2-D structures in solution were probed as follows [29]: 200 ng of transcripts dissolved at a 80 nM concentration in buffer D (20 mM Hepes-KOH, pH 7.9, 100 mM KCl, 0.2 mM EDTA pH 8.0, 0.5 mM DTT, 0.5 mM PMSF, 20% (vol/vol) glycerol) were renatured by 10 min heating at 65°C, followed by slow cooling at room temperature with the addition of 1 µl of 62.5 mM MgCl_2_ to a final concentration of 3.25 mM MgCl_2_. After a 10 min preincubation at room temperature, RNase T1 (0.02 or 0.0375 U/µl) or T2 (0.025 or 0.0375 U/µl) was added under conditions such that it cleaved single-stranded segments. V1 RNase (2.5×10^−3^ or 5×10^−3^ U/µl) was used to cleave double-stranded and stacked residues. DMS (1 µl of a 1/4 or 1/8 (V/V) DMS/EtOH solution) was employed to modify single-stranded A and C residues and CMCT (4 or 5 µl of a 180 mg/ml solution) to modify single-stranded U and to a lower extent G residues. Reactions were stopped as described in [29]. Cleavage and modification positions were identified by primer extension [29]. Stable secondary structures having the best fit with experimental data were identified with the Mfold software, version 8.1 [30]. Probing data were introduced as a constraint in the search.

### Steady-State Fluorescence Measurements

Fluorescence spectra were recorded at 4°C, with an excitation wavelength of 515 nm and scanning from 500 to 750 nm (excitation and emission bandwidth of 3 nm). The procedure used was derived from [24]. The RNA and Cy3-oligonucleotide were mixed at a 1∶1 molar ratio in 160 µl of 150 mM NaCl, 3.25 mM MgCl_2_, and 15 mM Na citrate (pH 7.0) to a final concentration (0.38 µM) superior to the Kd, incubated at 85°C for 5 min, and slowly cooled at room temperature for 15 min. After 4 h of incubation at 4°C, the yield of oligonucleotide association was determined by electrophoresis in a non-denaturing gel. Fluorescence in the gel was measured with a Typhoon (9410) Healthcare scanner. When a satisfying yield of association was detected, the emission of the Cy3-labeled complex was measured on a flux spectrofluorometer (SAFAS). Ten spectra were averaged. Then, the Cy5-labeled oligonucleotide was added at a 1∶1 molar ratio, and incubation carried out at 4°C for 4 h. Ten spectra were recorded and the Fluorescence Resonance Energy Transfer (FRET) for the Cy3–Cy5 pair was calculated taking into account the bound/unbound ratio of Cy3-oligonucleotide. Each FRET experiment was repeated three times using different batches of transcripts.

### Purification of RNP by MS2 Selection Affinity

The entire mouse A region and several fragments were cloned 3′ to a T7 promoter and 5′ to the MS2 tag present in plasmid pAdML3 [27],[31].

Nuclear extracts from undifferentiated female ES cells (LF2) were prepared according to [32], and dialyzed against buffer D. One hundred pmol of MS2-tagged RNAs were denatured, renatured, as described above, and incubated with a 5-fold molar excess of purified MS2-MBP fusion protein [31] at 4°C for 15 min. The RNA-MS2-MBP complexes formed were incubated with amylose beads (40 µl, GE Healthcare) equilibrated in buffer D for 2 h at 4°C. After three washes with 500 µl of buffer D, 1 mg of nuclear extract in 150 µl of buffer D containing 5 µM of yeast tRNAs was added. After 15 min of incubation at 4°C with constant agitation, three successive washes were performed in Buffer D and RNP complexes eluted by incubation with 80 µl of Buffer D containing 10 mM maltose (30 min at 4°C). Half of the eluted RNP complex formed with the entire A region was fractionated by 10% SDS-PAGE for mass spectrometry analyses. For all the purified RNP complexes, 10% of the eluted material was used for Western blot analysis performed according to [33].

### Mass Spectrometry Analysis

Each lane of the SDS-PAGE was cut into 2 mm sections, and proteins submitted to in-gel trypsin digestion. Analysis of extracted peptides was performed using nano-LC-MS-MS on a CapLC capillary LC system coupled to a QTOF2 mass spectrometer (Waters) according to standard protocols (Figure S8). The MS/MS data were analyzed using the MASCOT 2.2.0. algorithm (Matrix Science) for search against an in-house generated protein database composed of protein sequences of Rattus and Mus downloaded from UniprotKB http://beta.uniprot.org/ (August 07, 2008) and protein sequences of known contaminant proteins such as porcine trypsin and human keratins concatenated with reversed copies of all sequences. Spectra were searched with a mass tolerance of 0.3 Da for MS and MS/MS data, allowing a maximum of 1 missed cleavage with trypsin and with carbamidomethylation of cysteines, oxidation of methionines, and N-acetyl protein specified as variable modifications. Protein identifications were validated when one peptide had a Mascot ion score above 35. Evaluations were performed using the peptide validation software Scaffold (proteome Software).

### Immunoselection of RNP

RNA transcripts were dephosphorylated, 5′-end labelled with [γ-^32^P]ATP (3,000 Ci/mmol), purified and quantified according to [34]. About 70 pmol of the RNA were denatured, renatured as described above, and incubated with 30 µg of nuclear extract for 30 min at room temperature with constant agitation. About 40 µl of Protein G-sepharose beads suspension blocked with BSA (2 µg) and coated for 2 h at 4°C with 10 µl of each antibodies were incubated with the RNP complexes for 2 h at 4°C in 300 µl of immunoseletion buffer (150 mM NaCl, 10 mM Tris-HCl, pH 8.0, NP40 0.1%). Beads were washed three times for 10 min at 4°C with 750 µl of immunoselection buffer containing 0.5% NP40. RNAs were phenol extracted, ethanol precipitated, fractionated on 7% polyacrylamide gel, and analysed by autoradiography.

## Supporting Information

Figure S1 **Identification of enzymatic cleavage and chemical modification of the mouse A region by primer extension.** The A region of mouse Xist RNA was in vitro transcribed and renatured as described in Material and Methods, before being submitted to limited digestion with the T1, T2, or V1 RNases under the conditions described in Materials and Methods. Primer extension analyses were performed using oligonucleotides 3760 (A), 3973 (B), 3866 (C), 3758 (D), 3971 (E), or 3757 (F) (Table S1) as primers. The resulting cDNAs were fractionated by electrophoresis on 7% denaturing polyacrylamide gels. Lanes U, G, C, and A correspond to the sequencing ladder obtained with the corresponding primers. Lanes marked by Contr correspond to primer extension analysis of undigested RNA. Nucleotide numbering on the left side of the autoradiograms is calculated taking the first residue of mouse Xist RNA as residue 1. The position of the repeats is indicated by vertical bars on the right-hand side of the autoradiograms. Two different analyses of CMCT modifications by primer extension with oligonucleotide 3757 are illustrated in (F). The autoradiogram on the right side of the panel was exposed for a longer time.
(10.09 MB TIF)

Figure S2 **Representation of experimental data on a 2-D structure in which repeats form individual stem-loop structures.** Each of the seven repeats as well as the eighth half repeat in the mouse A region were folded into a unique stem-loop structure with an internal loop. T1, T2, and V1 RNase cleavages are represented by arrows surmounted by circles, triangles, and squares, respectively. Nucleotides modified by DMS or CMCT are circled. The colours of circles and arrows indicate the modification and cleavage yields, with red, yellow, and green corresponding, respectively, to strong, medium, and low modification or cleavage. The V1 RNase cleavages and chemical modifications that cannot be explained by this secondary structure model are circled in blue.
(0.73 MB TIF)

Figure S3 **Identification of enzymatic cleavage and chemical modification in human A region by primer extension analysis.** The A region of human XIST RNA was treated as described for the mouse A region in the legend to Figure 1 of supporting data, except that the primers used for extension analyses were oligonucleotides 4563 (A), 4564 (B), 4622 (C), 4565 (D), and 4242 (E) (Table S1).
(7.04 MB TIF)

Figure S4 **Representation of experimental data on the possible structure 1 of human Xist A region.** In this model, stem-loop structures involve two successive repeats. The repeats are indicated by red lines and are numbered from 1 to 9. Representation of chemical and enzymatic data is as in Figures 2 and 3. The free energies of each stem-loop structure at 0°C and in 3 M NaCl were calculated with the M-fold software.
(0.74 MB TIF)

Figure S5 **Conservation of sequences surrounding the repeats in vertebrate A regions.** Sequence alignment of the mouse, human, Orangutan, baboon, lemur, dog, rabbit, cow, horse, and elephant A regions illustrating the degree of species conservation. Identical nts are indicated in red. Repeats are numbered from 1 to 9 and shown as red rectangles, mouse (gi|37704378|ref|NR_001463.2|), human (gi|340393|gb|M97168.1|), Orangutan (by http://www.ensembl.org/index.html, 292L3-1,185272), baboon (by http://www.ensembl.org/index.html, 157F22-1,190936), lemur (by http://www.ensembl.org/index.html, 176F24-1,134555), dog [by http://genome.ucsc.edu/,(canFam2) assembly canFam1_dna range = chrX:60100000–60735000)], rabbit (gi|1575009|gb|U50910.1|OCU50910), cow (gi|10181229|gb|AF104906.5|), horse (gi|1575005|gb|U50911.1|), and elephant (BROADE1:scaffold_119260:3220:3899:−1 ENSEMBL).
(0.20 MB DOC)

Figure S6 **The possibility to form four-repeats stem-loop (SLS1 and SLS3) structure is conserved in vertebrates.** SLS1 and SLS3 in mouse, dog, human, rabbit, and elephant are folded according to the mouse SLS1 structure (Model 3). The name of each species is indicated below the structure. Sequence variations compared to the mouse A sequence are indicated in green.
(1.21 MB TIF)

Figure S7 **Control FRET experiment performed with two oligonucleotides bordering one helix.** (A) Schematic presentation of the transcripts used in the control experiment. (B) Fluorescence spectra obtained with the donor P1 oligonucleotide bound to naked 2R/RNA (green curve) and with oligonucleotides P1/P3′ bound to the RNA (violin curve). See legend in Figure 8 for details.
(0.18 MB TIF)

Figure S8 **Identification of components of the PRC2 complex by mass spectrometry.** Peptides that served for each protein identification are summarized in a table with corresponding MS/MS spectra. (A) Identification of Enhancer of zeste homolog 2 (Ezh2). (B) Identification of Polycomb protein Suz12 (Suz12). (C) Identification of Retinoblastoma binding protein 4 (RbAp46). (D) Identification of Retinoblastoma binding protein 7 (RbAp48). (E) Detailed standard protocols for proteomic analyses.
(3.01 MB DOC)

Figure S9 **Folding capacities of the synthetic A region sequences whose activity was evaluated in** **[11]** **.** Sequence XR corresponds to the positive control, XSR: replacement of GCCCAUCGCGGG by CGGGAUCGGCCC; XCR have small U-rich spacer regions. XS1: deletion of the GG dinucleotides in the second element of each repetition, XNX: replacement of GGGCAUCGGGGC by GCGCAUCGGAGC. Silencing properties of Xist RNA containing these synthetic variants A region are indicated in the right-hand side panel of the Table S1.
(0.54 MB TIF)

Table S1 **Oligonucleotides used in this study.** The name, sequence, and utilization of each oligonucleotide are given. Nucleotide positions of A region are numbered according to the genBank accession no. gi|37704378|ref|NR_001463.2| (Mouse Xist gene) [2] and no. gi|340393|gb|M97168.1| (Human *XIST* gene) [5]. Restriction sites and fluorescent dyes introduced by the oligonucleotides are indicated.
(0.05 MB DOC)

## Abbreviations

CMCT: N-cyclohexyl-N0-(2-morpholinoethyl)-carbodiimide metho-p-toluolsulfonate
DMS: dimethylsulfate
Eed: embryonic ectoderm development
ES cell: embryonic stem cell
Ezh2: enhancer of zeste homolog 2
FRET: Förster resonance energy transfer
HIV: human immunodeficiency virus
HOTAIR: HOX antisense intergenic RNA
Lnx3: ligand of numb-protein X 3
NMR: nuclear magnetic resonance
PRC2: polycomb group protein 2
PTB: polypyrimidine tract-binding protein
Rbap46–48: retinoblastoma-binding protein p46–p48
RNP: ribonucleoprotein particle
RRM: RNA recognition motif
Suz12: suppressor of zeste 12 protein homolog
XCI: X chromosome inactivation
Xist: X (inactive)-specific transcript
