# Supplementary figures and images for: 2-D Structure of the A Region of Xist RNA and Its Implication for PRC2 Association
Source: PLoS Biol. 2010 Jan 5;8(1):e1000276. doi: 10.1371/journal.pbio.1000276 (PMC2796953; doi:10.1371/journal.pbio.1000276)

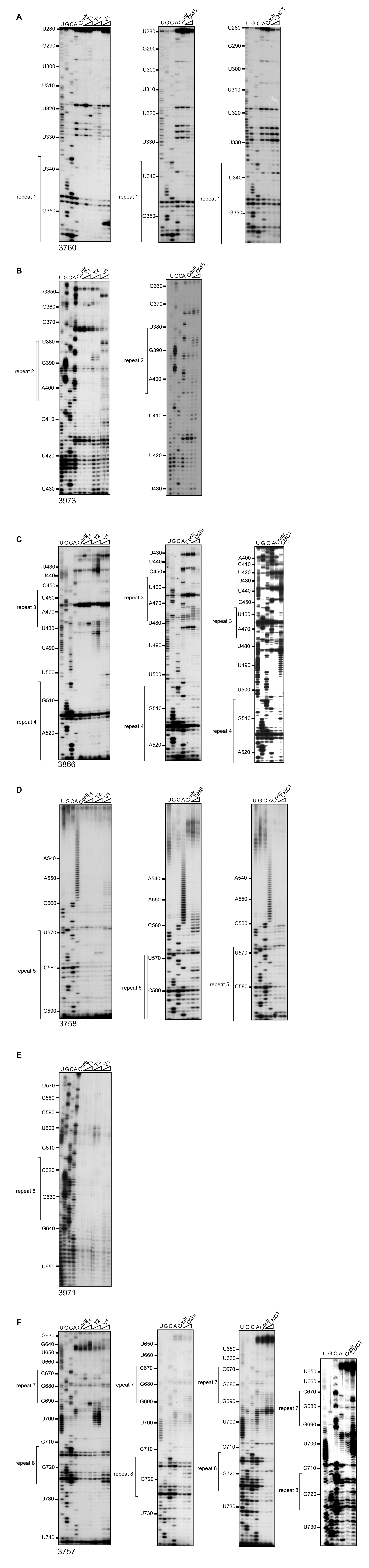

Supplement: Figure S1 — Identification of enzymatic cleavage and chemical modification of the mouse A region by primer extension. The A region of mouse Xist RNA was in vitro transcribed and renatured as described in Material and Methods, before being submitted to limited digestion with the T1, T2, or V1 RNases under the conditions described in Materials and Methods. Primer extension analyses were performed using oligonucleotides 3760 (A), 3973 (B), 3866 (C), 3758 (D), 3971 (E), or 3757 (F) (Table S1) as primers. The resulting cDNAs were fractionated by electrophoresis on 7% denaturing polyacrylamide gels. Lanes U, G, C, and A correspond to the sequencing ladder obtained with the corresponding primers. Lanes marked by Contr correspond to primer extension analysis of undigested RNA. Nucleotide numbering on the left side of the autoradiograms is calculated taking the first residue of mouse Xist RNA as residue 1. The position of the repeats is indicated by vertical bars on the right-hand side of the autoradiograms. Two different analyses of CMCT modifications by primer extension with oligonucleotide 3757 are illustrated in (F). The autoradiogram on the right side of the panel was exposed for a longer time. (10.09 MB TIF) [file pbio.1000276.s001.tif]

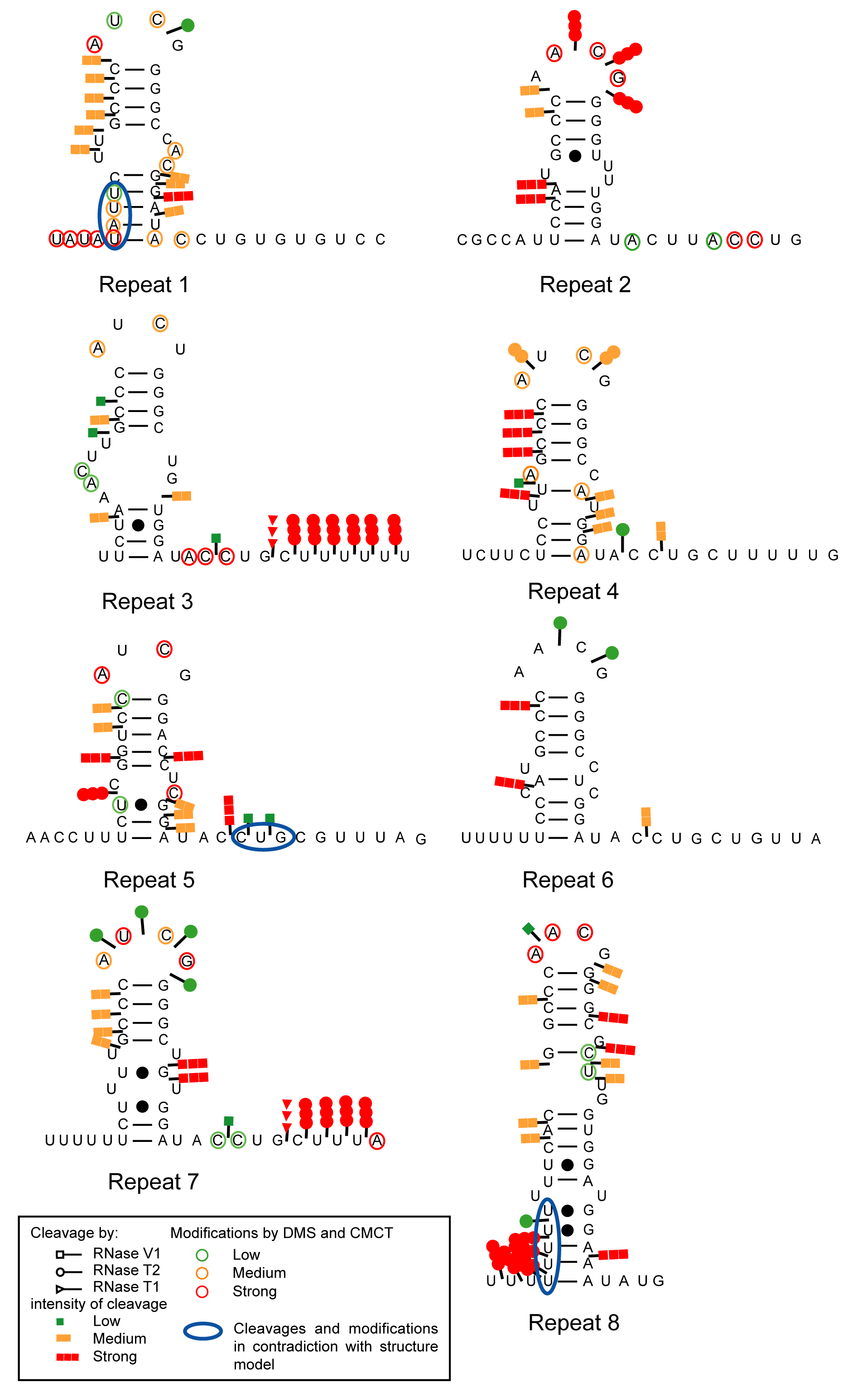

Supplement: Figure S2 — Representation of experimental data on a 2-D structure in which repeats form individual stem-loop structures. Each of the seven repeats as well as the eighth half repeat in the mouse A region were folded into a unique stem-loop structure with an internal loop. T1, T2, and V1 RNase cleavages are represented by arrows surmounted by circles, triangles, and squares, respectively. Nucleotides modified by DMS or CMCT are circled. The colours of circles and arrows indicate the modification and cleavage yields, with red, yellow, and green corresponding, respectively, to strong, medium, and low modification or cleavage. The V1 RNase cleavages and chemical modifications that cannot be explained by this secondary structure model are circled in blue. (0.73 MB TIF) [file pbio.1000276.s002.tif]

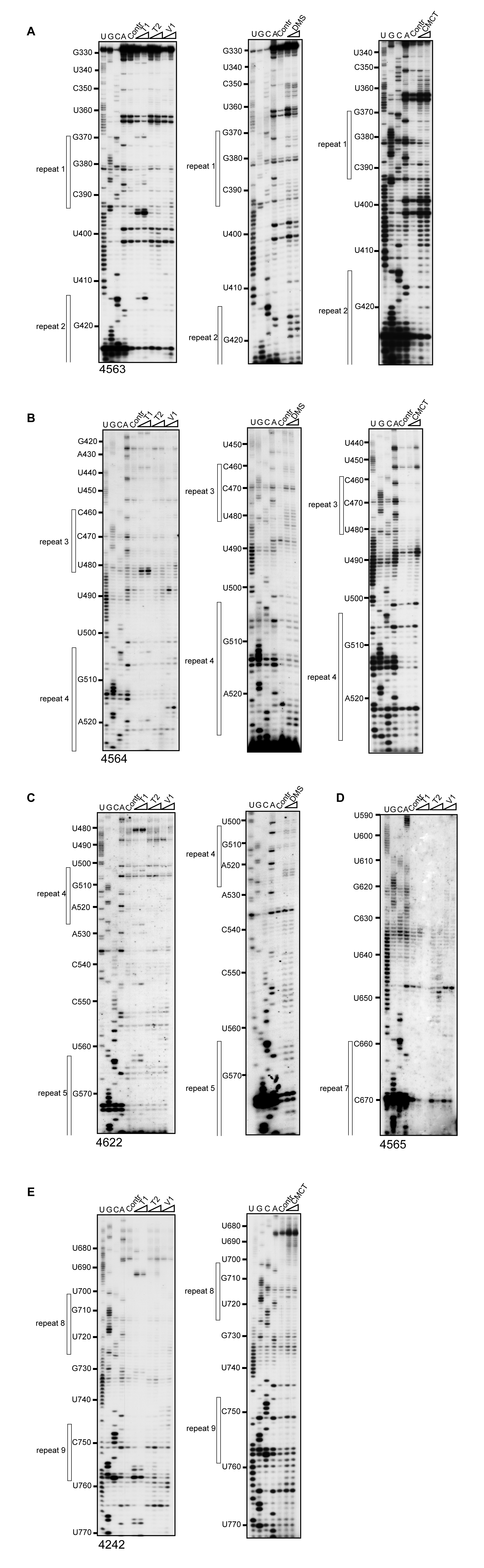

Supplement: Figure S3 — Identification of enzymatic cleavage and chemical modification in human A region by primer extension analysis. The A region of human XIST RNA was treated as described for the mouse A region in the legend to Figure 1 of supporting data, except that the primers used for extension analyses were oligonucleotides 4563 (A), 4564 (B), 4622 (C), 4565 (D), and 4242 (E) (Table S1). (7.04 MB TIF) [file pbio.1000276.s003.tif]

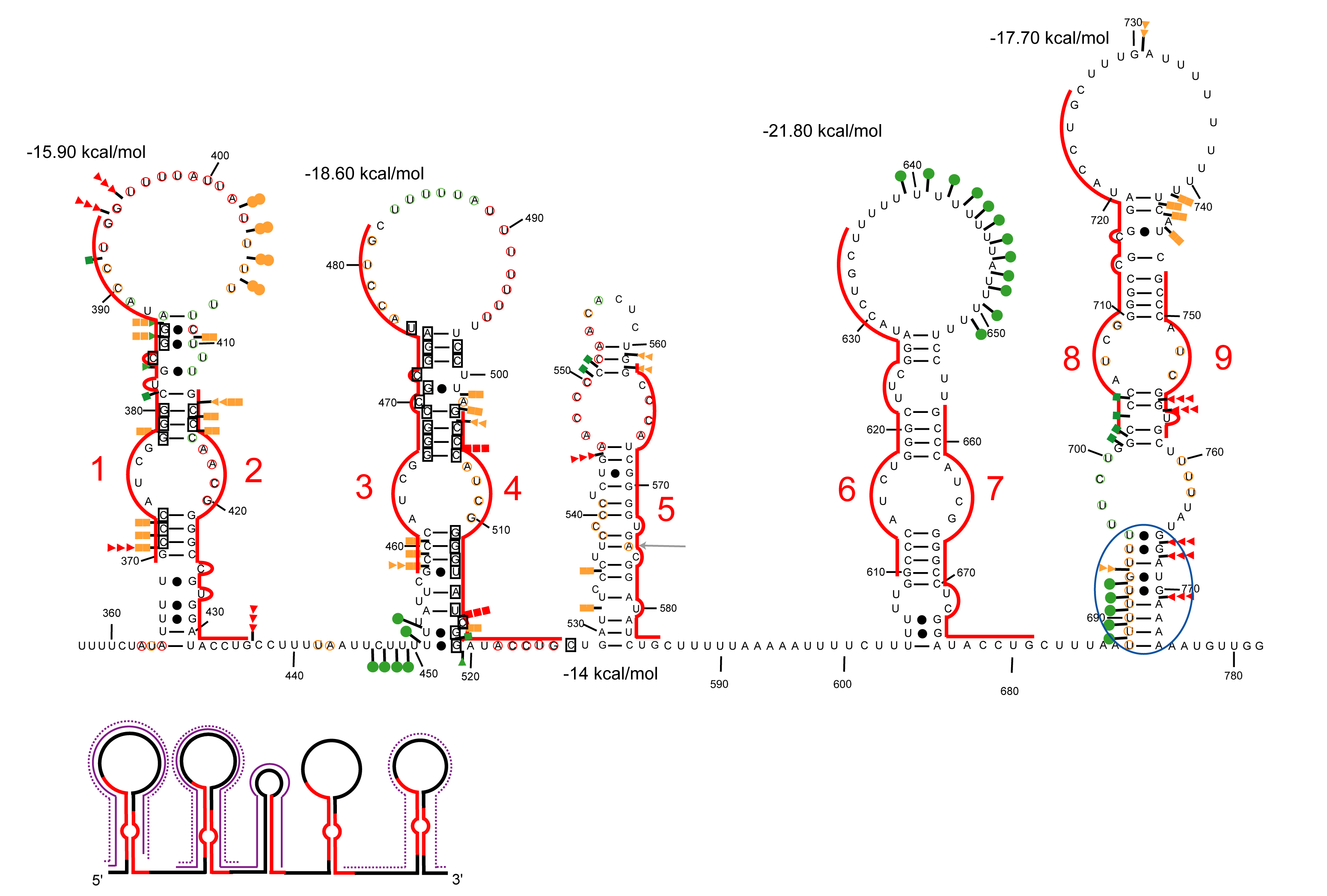

Supplement: Figure S4 — Representation of experimental data on the possible structure 1 of human Xist A region. In this model, stem-loop structures involve two successive repeats. The repeats are indicated by red lines and are numbered from 1 to 9. Representation of chemical and enzymatic data is as in Figures 2 and 3. The free energies of each stem-loop structure at 0°C and in 3 M NaCl were calculated with the M-fold software. (0.74 MB TIF) [file pbio.1000276.s004.tif]

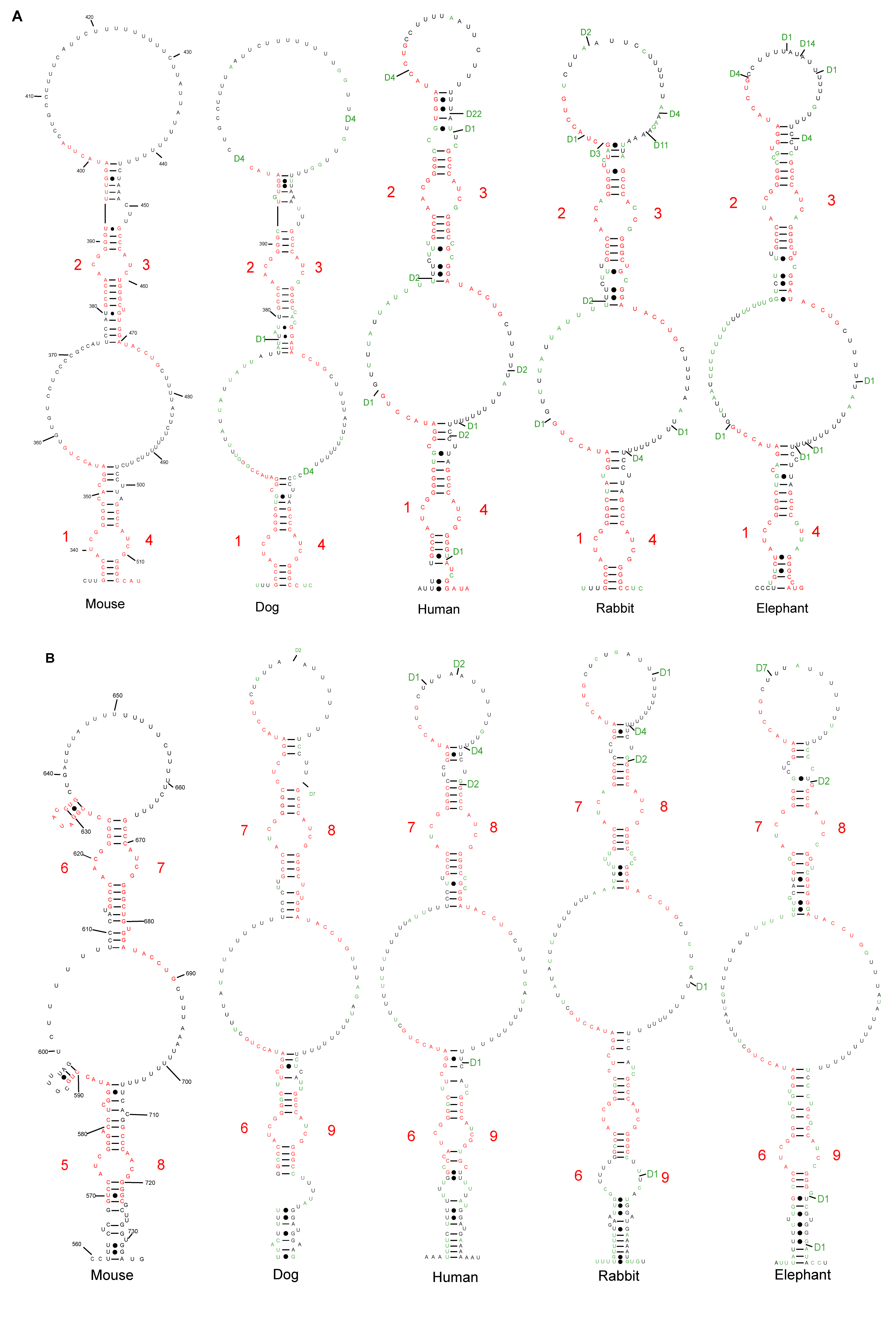

Supplement: Figure S6 — The possibility to form four-repeats stem-loop (SLS1 and SLS3) structure is conserved in vertebrates. SLS1 and SLS3 in mouse, dog, human, rabbit, and elephant are folded according to the mouse SLS1 structure (Model 3). The name of each species is indicated below the structure. Sequence variations compared to the mouse A sequence are indicated in green. (1.21 MB TIF) [file pbio.1000276.s006.tif]

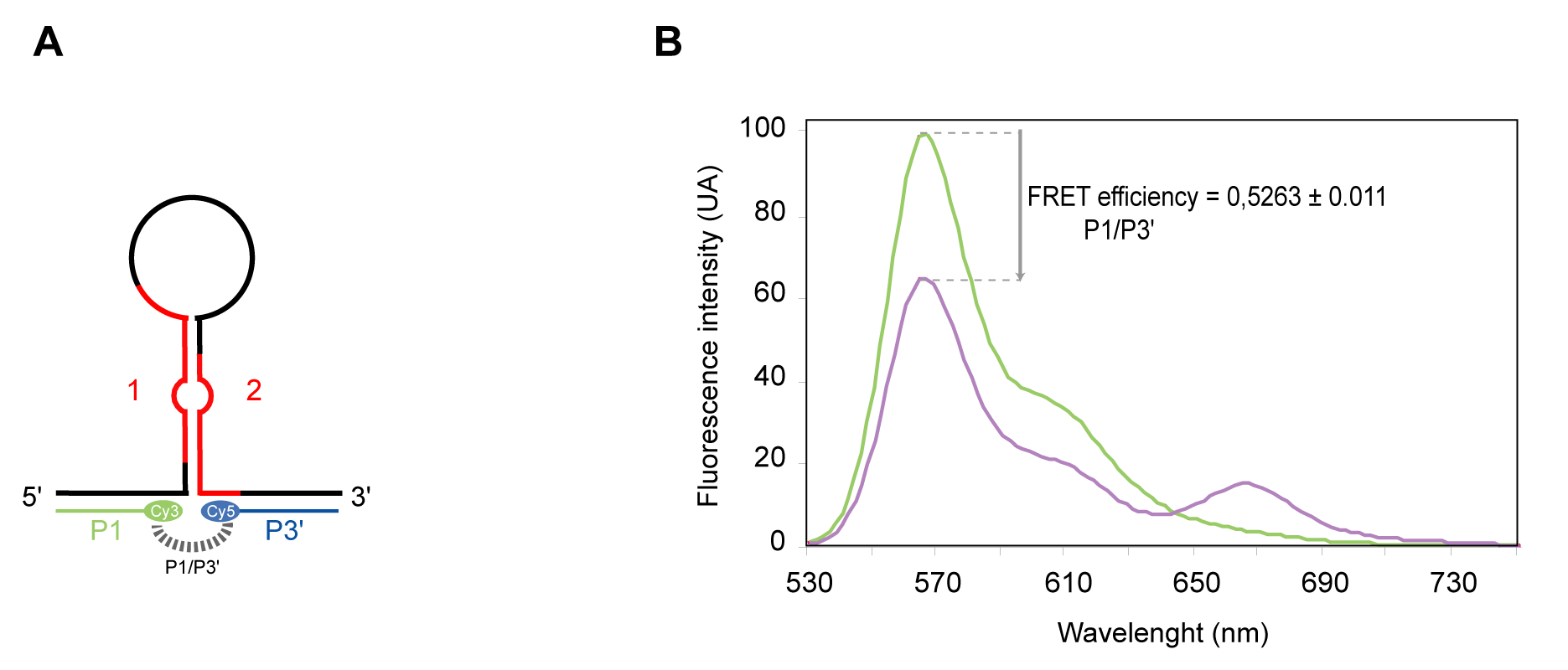

Supplement: Figure S7 — Control FRET experiment performed with two oligonucleotides bordering one helix. (A) Schematic presentation of the transcripts used in the control experiment. (B) Fluorescence spectra obtained with the donor P1 oligonucleotide bound to naked 2R/RNA (green curve) and with oligonucleotides P1/P3′ bound to the RNA (violin curve). See legend in Figure 8 for details. (0.18 MB TIF) [file pbio.1000276.s007.tif]

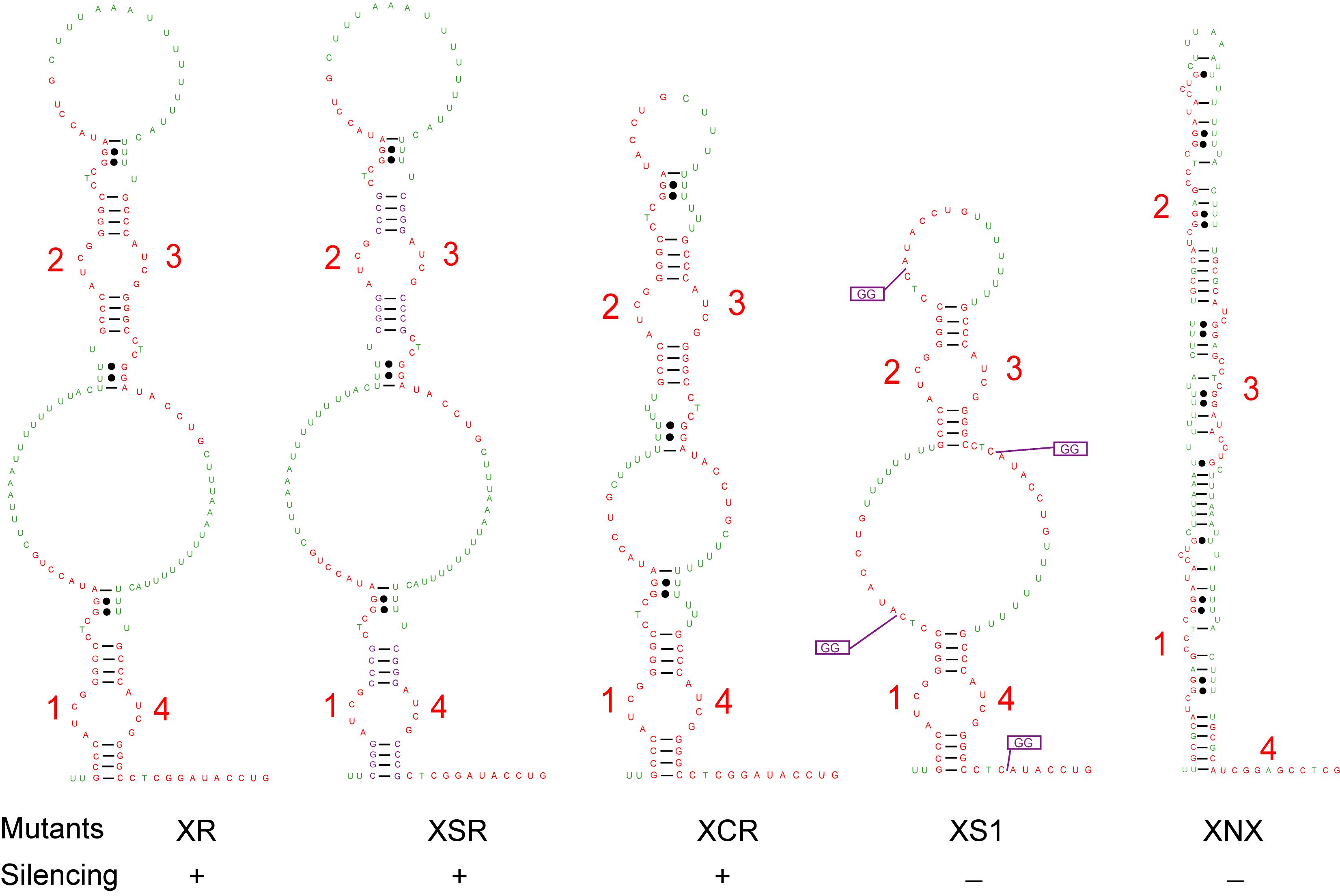

Supplement: Figure S9 — Folding capacities of the synthetic A region sequences whose activity was evaluated in [11] . Sequence XR corresponds to the positive control, XSR: replacement of GCCCAUCGCGGG by CGGGAUCGGCCC; XCR have small U-rich spacer regions. XS1: deletion of the GG dinucleotides in the second element of each repetition, XNX: replacement of GGGCAUCGGGGC by GCGCAUCGGAGC. Silencing properties of Xist RNA containing these synthetic variants A region are indicated in the right-hand side panel of the Table S1. (0.54 MB TIF) [file pbio.1000276.s009.tif]
