# Supplementary material for: 2-D Structure of the A Region of Xist RNA and Its Implication for PRC2 Association
Source: PLoS Biol. 2010 Jan 5;8(1):e1000276. doi: 10.1371/journal.pbio.1000276 (PMC2796953; doi:10.1371/journal.pbio.1000276)
Supplement: Figure S5 — Conservation of sequences surrounding the repeats in vertebrate A regions. Sequence alignment of the mouse, human, Orangutan, baboon, lemur, dog, rabbit, cow, horse, and elephant A regions illustrating the degree of species conservation. Identical nts are indicated in red. Repeats are numbered from 1 to 9 and shown as red rectangles, mouse (gi|37704378|ref|NR_001463.2|), human (gi|340393|gb|M97168.1|), Orangutan (by http://www.ensembl.org/index.html, 292L3-1,185272), baboon (by http://www.ensembl.org/index.html, 157F22-1,190936), lemur (by http://www.ensembl.org/index.html, 176F24-1,134555), dog [by http://genome.ucsc.edu/,(canFam2) assembly canFam1_dna range = chrX:60100000–60735000)], rabbit (gi|1575009|gb|U50910.1|OCU50910), cow (gi|10181229|gb|AF104906.5|), horse (gi|1575005|gb|U50911.1|), and elephant (BROADE1:scaffold_119260:3220:3899:−1 ENSEMBL). (0.20 MB DOC) [file pbio.1000276.s005.rtf]

Mouse        -------------- ---------- --------CC TTTCTTTCAT TGTTTATATA TTC-TTGCCC ATCGGGGCCA CGGATACCTG TGTGTCCTCC CC-------- ---------- ---------- -----GCCAT TCCATG 
Human        ----TCTCTGTTTT TTGTGGATCA GTTTTTTACT CTTCCACTCT CTTTTCTATA TT--TTGCCC ATCGGGGCTG CGGATACCTG GTTTTATTAT ---------- ---------- ---------- ------TTTT TCTTTG 
OrangUtan    -------------- ---TGGATCA GTTTTCTACT CTTCCACTCT CTTTTATAT- -T--TTGCCC ATCGGGGCTG CGGATACCTG GTTTTATTAT ---------- ---------- ---------- ------TTTT TCTTTG 
Baboon       -------------- ---------- ---------- ---------- ---------- -T--TTGCCC ATCGGGGCTG CGGATACCTG GTTTTATTAT ---------- ---------- ---------- ------TTTT TCTTTG 
Lemur        ----TCACTTTTTT GTATCATTTA GTATTCTTAG TGTATACTGT -TTTTGTATA TT--TGGCCC ATCGGGGCTG CGGATACCTG GTTTCATTAT TG-------- ---------- ---------- -----TTTTT TCTTTG 
Dog          -------------- --------GT TTGCTTTTCT TTTTCCTTCT TTTTTCAATA TT--TTGCCC ATCGGGGCTG CGGATACCGG GTTTTATTAT ---------- ---------- ---------- --TATTATTA TTATTG 
Rabbit       ----TGACTCCTCT GTTTTGGGGG TTCAGATTTC TTTTCTCCTA CATTTCCATA TT--TTGCCC ATCGGGGCTA TGGATACCTG GTTTTATTAT ---------- ---------- ---------- ------TTTT TCTTTG 
Cow          -------------- ---------- ---------- -----CTCCT TTTCTATATT AT--TTGCCC ATCGCGGCTG TGGATACCTG GTTTTAGTGT GTATATAGAT AGGTAGATAG ATATATTATT ATTGTATTAT TTTTTA 
Horse        -------------- ---------- ---------- ---------- -----ATATT AT--TTGCCC ATCGCGGCTG TGGATACCTG GTTTTAGTGT GTATATAGAT AGGTAGATAG ATATATTATT ATTGTATTAT TTTTTA 
Elephant     -------------- ---------- ---------- ---------- -------TTG TCCCTTGTCT ATCCGGGCTG CAGATACCTG GTTTAATTTT TTTTTT---- ---------- ---------- --TTTTTTGG TCTTTG 


Mouse        CCCAACGGGG TTTTGGATAC TTACCTGCCT TTTCATTCTT TTTTTTTCTT ATTA--TTTT TTTTTCTAAA CTTGCCCATC TGGGCTGTGG ATACCTGCTT TTATTCTTTT TTTCTTCTCC TTAGCCCATC GGGGCCATGG 
Human        CCCAACGGGG CCGTGGATAC ----CTGCCT TTTAATTCTT TTTT------ ---------- ---------A TTCGCCCATC GGGGCCGCGG ATACCTGCTT TTTATTTTTT TTT-----CC TTAGCCCATC GGGGTATCGG 
OrangUtan    CCCAACGGGG CCGTGGATAC ----CTGCCT TTTAATTCTT TTTT------ ---------- ---------A TTCGCCCATC GGGGCCGCGG ATACCTGCTT TTTATTTTTT TTTTT---CC TTAGCCCATC GGGGTATCGG 
Baboon       CCCAACGGGG CTGTGGATAC ----CTACCT TTTAATTCCT TTTT------ ---------- ---------A TTCGCCCATC GGGGCCGCGG ATACCTGCTT TTAATTTTTT TTTTTTT-CC TTAGCCCATC GGGGCATCGG 
Lemur        CCCAACGGGG CTGTGGATAG ----CTGCTT TTTAATTCTT TTTCTT---- ---------- --TTTCAAAA TTTGCCTATC GGGGCCGTGG ATACCTGCTT TTTTTTTTTT TTTT----CC TTAGCCCATC GGGGCAGCGG 
Dog          CCCAACGGGG CTGTGGATAC ----CTGCCT TTTAATTCTT TTTTTTGGTT ------TGTT GGTTTTTAAA TTTGCCCATC GGGGCCACGG ATACCTGCTT TTATTTTTTT TTCC----CC TTAGCCCATC GGGGCCTCGG 
Rabbit       CCCAACAGGG TTCTAGCTAC ----CTG--T CTTAATTCCT TTTTTAAAG- ---------- ------AAAA TTAGCCCACC GGGGCTGCGG ATACCTGCTT TTAATTTTTT TTT-----CC TTAGCCCATC GGGGCCTCGG 
Cow          CCCAACGGGG TCATGGATAC ----CTGCCT TTTATTTTAT TTTTTT---- ---------- ------TTAA TTTGCCCATC GGGGCCACGG ATACCTGCTT TTAATTTTTT TTTTCCC-CC TTAGCCCATC GGGGCCTCGG 
Horse        CCCAACGGGG TCATGGATAC ----CTGCCT TTTATTTTAT TTTTTT---- ---------- ------TTAA TTTGCCCATC GGGGCCACGG ATACCTGCTT TTAATTTTTT TTTTCCC-CC TTAGCCCATC GGGGCCTCGG 
Elephant     CCCATCGGGG CCGTGGATAC ----CTGCCT TTTATATTTT TTGTTTT--- ---------- ---------C CTCGCCCATC AGGGCTGCGG ATACCTGCTT TTTAATTTTT TTTTT---CC TTAGCCCGTT AGGGCCATGG 


Mouse        ATACCTGCTT TTT------- ---------- ---------- ------GTAA AAAAAAAAAA AA----A--- ---------- ---AAAAAAA ACCTTTCTCG G--------- ---TCCATCG GGACCTCGGA TACCTGCGTT 
Human        ATACCTGCTG ATTCCCTTCC CCTCTGAACC CCCAACACTC TGGCCCATCG GGGTGACGGA TATCTGC--- --------TT TTTAAAAATT TTCTTTTTTT G--------- --GCCCATCG GGGCTTCGGA TACCTGCTTT 
OrangUtan    ATACCTGCTG ATTCCCTTCC CCTCTGAACC CCCAACACTC TGGCCCATCG GGGTGACGGA TACCTGC--- --------TT TTTAAAAATT TTCTTTTTTG G--------- ---CCCATCG GGGCTTCGGA TACCTGCTTT 
Baboon       ATACCTGCTG ATTCCCTTCC CCTCTGACCC CCCAACACTC TGGCCCATCG GGGTGGCGGA TACCTGC--- --------TT TTTAAAAATT TTGTTTTTTT G--------- --GCCCATCG GGGCTTCGGA TACCTGCTTT 
Lemur        ATACTTGCTG TGTCCACCCG CCCCTCCCCC CACTCCTCGC TGGCTGATCG GGGCAACGGA CACCCGC--- --------TT TTTAAAAATA TTTGTTTTTG G--------- ---CCCATCG GGGCCTCGGA TACCTGCTTT 
Dog          ATACCTGCTG TGCCCC---C CC------CC CCCAACTCCC TGGCCCATCG GGGCAATGGA TACCTGC--- ---------- TTAAAAAAAA TTACTTTTG- ---------- --GCCCATCG GGGCTTCGGA TACCTGCTTT 
Rabbit       ATACCTGCAG TGCCCC---- CTTTTGCCCC CCAAAT--TC TGGCCCATCG GGGCAACGGA TACCTGTTTT TGTGGTTTTT TTTGTTTTGT TTTGTTTTTG AATTGGCTTT TGGCCCATCG GGGCCTCGGA TACCTGCTTA 
Cow          ATACCTGCTG TGTACC---C CCTCT--CTC CCTAAC---C TGGCCCATCG GGGCAATGGA TACCTGCCTC T-------TT TTTAAATGTG TTGTTTTTTT TTTTTTTTCC TTGCCCATCG GGGCCTCGGA TACCTGCTTT 
Horse        ATACCTGCTG TGTACC---C CCTCT--CTC CCTAAC---C TGGCCCATCG GGGCAATGGA TACCTGCCTC T-------TT TTTAAATGTG TTGTTTTTTT TTTTTTTTCC TTGCCCATCG GGGCCTCGGA TACCTGCTTT 
Elephant     ATAACTGCTG TGTCCC---- CCCCCCCCCA CCCAAC-CAC TGGCCCATCG GGACAACGGA GACCTGTTCC T------TTT TTCCCATAAT TTATTTTTTG G--------- ---CCCATCG GGGCTGTGGA TACCTGCTTT 


Mouse        TAGT-CTTTT T-----TTCC CATGCCCAAC GGGGCCTCGG ATACCTGCTG TTATTATTTT TTTTTCTTTT TCTTTTGCCC ATCGGGGCTG TGG-ATACCT GCTTTAAATT TTTTTTTT-C ACGGC-CCAA CGGGGCGCTT 
Human        TTTT-TTTTT TAT--TTTTC CTTGCCCATC GGGGCCTCGG ATACCTGCTT TAATT----- --TTTG--TT TTTCTGGCCC ATCGGGGCCG CGG-ATACCT GCTTTGATTT TTTTTTTT-C ATCGC-CCAT CGGTGCTTTT 
OrangUtan    TTTT-TTTTT TTTATTTTTC CTTGCCCATC GGGGCCTCGG ATACCTGCTT TAATT----- --TTG---TT TTTCTGGCCC ATCGGGGCCG CGG-ATACCT GCTTTGATTT TTTTTT---C ATCGC-CCAT CGGTGCTTTT 
Baboon       TATT-TTTTA T----TTTTC CTTGCCCATC GGGGCCTCGG ATACCTGCTT TAATT----- --TTTG--TT TTTCTGGCCC ATCGGGGCCG CGG-ATACCT GCTTTGATTT TTTTTTTTTC ATCGC-CCGT CGGTGCTTTT 
Lemur        TATT-ATTTT ------TTTC CTTGCCCATC GGGGCCTCGG ATACCTGCTT TTATT----- --ATTT--TT TTCCTTGCCC ATCGGGGCCT CGG-ATACCT GCTCTAATTT TTTTTTTC-C ATCGC-CCAG CGGGGCTTTA 
Dog          TATT-TTTTT T-----TTCC CTTGCCCATC GGGGCCTCGG ATACCTGCTT TTAAT----- --TTT---TT TTCCTTGCCC ATCGGGGCTG TGG-ATACCT GTTTAGATTT TTTTTTTCTC ATTGC-CCAT CGGGGCCTTT 
Rabbit       TATT-TTTTT TTTA-AATTT TTTGCCCATC AGGGCCTCGG ATACCTGCTC TGATT----- --TTTTTTTT TTTCTGGCCC ATCGGGGCCC CGG-ATACCT GCTCTGATTT TTTTTTTC-C ATCGC-CCAT CGGGGCCTTT 
Cow          AATT-TTTT- ------TTTT CTTGCCCATC GGGGCCTCGG ATACCTACTT TAATT----- --TTT----T TTCCTTGCCC ATCGGGGCCG CGG-ATACCT GCTTAGATTT TTGTTTTA-C ACCAC-CCAT CGGGGCTTTA 
Horse        AATT-TTTT- ------TTTT CTTGCCCATC GGGGCCTCGG ATACCTACTT TAATT----- --TTT----T TTCCTTGCCC ATCGGGACCG CGG-ACACCT GCTTAGATTT TTGTTTTA-C ACCAC-CCAT CGGGGCTTTA 
Elephant     TATTGTTTTT TTTTTTTTTG CATGCGCATC GGGGGCTCGG ATACCTGCTT TATTT----- --TTTT--TT TTCCCTGCCC ATCCGGGTCG TGGGATACCT GGTTTATATT TTTTTTTTTC CCTGCGCCAT CCGGGGTCGT 
