# Supplementary material for: 2-D Structure of the A Region of Xist RNA and Its Implication for PRC2 Association
Source: PLoS Biol. 2010 Jan 5;8(1):e1000276. doi: 10.1371/journal.pbio.1000276 (PMC2796953; doi:10.1371/journal.pbio.1000276)
Supplement: Figure S8 — Identification of components of the PRC2 complex by mass spectrometry. Peptides that served for each protein identification are summarized in a table with corresponding MS/MS spectra. (A) Identification of Enhancer of zeste homolog 2 (Ezh2). (B) Identification of Polycomb protein Suz12 (Suz12). (C) Identification of Retinoblastoma binding protein 4 (RbAp46). (D) Identification of Retinoblastoma binding protein 7 (RbAp48). (E) Detailed standard protocols for proteomic analyses. (3.01 MB DOC) [file pbio.1000276.s008.rtf]

A  Identification of Enhancer of zeste homolog 2 (EZH2), 

B  Identification of Polycomb protein suz12 (SUZ12), 


C  Identification of Retinoblastoma binding protein 4 (RbAp46), 


D  Identification of Retinoblastoma binding protein 7 (RbAp48). 


E  Detailed standard protocols for proteomic analyses.

In gel digestion: 
In gel digestion was performed with an automated protein digestion system, MassPrep Station (Waters, Manchester, UK). The gel plugs were washed twice with 50 µL of 25 mM ammonium hydrogen carbonate (NH4HCO3) and 50 µL of acetonitrile. The cysteine residue were reduced by 50 µL of 10 mM dithiothreitol at 57°C and alkylated by 50 µL of 55 mM iodoacetamide. After dehydration with acetonitrile, the proteins were cleaved in gel with 20 µL of 12.5 ng/µL of modified porcine trypsin (Promega, Madison, WI, USA) in 25 mM NH4HCO3. The digestion was performed overnight at room temperature. The generated peptides were extracted with 60% acetonitrile in 1% acid formic.

nanoLC-MS/MS: 
From each sample, 6.4 µL were loaded on a precolumn, before chromatographic separation on a C18 column (LC Packings C18, 75 mm id, 150 mm length). The gradient was generated by the CapLC at a flow rate of 200 nL/min. The gradient profile consisted of a linear one from 90% of a water solution acidified by 0.1% HCOOH vol/vol (solution A), to 40% of a solution of CH3CN acidified by 0.1% HCOOH vol/vol (solution B) in 30 min, followed by a second gradient ramp to 75% of B in 1 min. Data acquisition was piloted by MassLynx software V4.0 (Waters). Calibration was performed using adducts of 0.1% phosphoric acid (Acros, NJ, USA) with a scan range from m/z 50 to 1800. Automatic switching between MS and MS/MS modes was used. The internal parameters of Q-TOF II were set as follows. The electrospray capillary voltage was set to 3.5 kV, the cone voltage set to 35 V, and the source temperature set to 90°C. The MS survey scan was m/z 300–1500 with a scan time of 1 s and an interscan time of 0.1 s. When the peak intensity rose above a threshold of 15 counts/s, tandem mass spectra were acquired. Normalized collision energies for peptide fragmentation were set using the charge-state recognition files for 1+, 2+, and 3+ peptide ions. The scan range for MS/MS acquisition was from m/z 50 to 2000 with a scan time of 1 s and an interscan time of 0.1 s. Fragmentation was performed using argon as the collision gas and with a collision energy profile optimized for various mass ranges of precursor ions. Data processing was done automatically with the ProteinLynx Process (Micromass) module.
