# Supplementary material for: 2-D Structure of the A Region of Xist RNA and Its Implication for PRC2 Association
Source: PLoS Biol. 2010 Jan 5;8(1):e1000276. doi: 10.1371/journal.pbio.1000276 (PMC2796953; doi:10.1371/journal.pbio.1000276)
Supplement: Table S1 — Oligonucleotides used in this study. The name, sequence, and utilization of each oligonucleotide are given. Nucleotide positions of A region are numbered according to the genBank accession no. gi|37704378|ref|NR_001463.2| (Mouse Xist gene) [2] and no. gi|340393|gb|M97168.1| (Human XIST gene) [5]. Restriction sites and fluorescent dyes introduced by the oligonucleotides are indicated. (0.05 MB DOC) [file pbio.1000276.s010.doc]

| Primer name | Sequence (5'-3') | Positions | Characteristics |
| --- | --- | --- | --- |
| Ampification for RNA preparation | |  |  |
| O-3755 | AAGCTTAAATTTAATACGACTCACTATAGGGCTTGCTCCAGCCATGTT | 1-20 | HindIII |
| O-3754 | TCTAGACGCGCAACACCGCACACTAA | 1117-1137 | XbaI |
| O-3756 | AAGCTTAAATTTAATACGACTCACTATAGGGTTGATACTTGTGTGTGTA | 277-297 | HindIII |
| O-3757 | TCTAGAGCAATAACTCACAAAACCAT | 741-760 | XbaI |
| O-4240 | AAGCTTAAATTTAATACGACTCACTATAGGGATCAGTTTTTTACTCTTC | 330-350 | HindIII |
| O-4241 | TCTAGAACAACCCACAAAACCAACA | 776-796 | XbaI |
| O-4567 | TCTAGACCAGAGTGTTGGGGGTTCAG | 542-562 | XbaI |
| O-4568 | AAGCTTAAATTTAATACGACTCACTATAGGACTCACTATAGGGAACCCCCAACACTCTGGCC | 545-565 | HindIII |
| 2D structure of Mouse A region | |  |  |
| O-3757 | TCTAGAGCAATAACTCACAAAACCAT | 741-760 | XbaI |
| O-3758 | TGTGAAAAAAAGACTAAACGC | 592-612 |  |
| O-3760 | ATGGAATGGCGGGGAGGACA | 361-381 |  |
| O-3866 | TTTTTTTTTTTTTTTTTTACAAAAAG | 527-552 |  |
| O-3971 | AGCCCCGATGGGCAAAAGA | 661-679 |  |
| O-3973 | GATGGGCAAGTTTAGA | 444-460 |  |
| 2D structure of Human A region | |  |  |
| O-4242 | ACAACCCACAAAACCAACAT | 776-796 |  |
| O-4563 | AAAAAGAATTAAAAGGCAGG | 432-452 |  |
| O-4564 | GGGTTCAGAGGGGAAGGGAA | 530-551 |  |
| O-4565 | AAAAATAAAAAAAAAAAAAG | 635-655 |  |
| O-4622 | AGAAAATTTTTAAAAAGCAG | 582-602 |  |
| FRET experiments | |  |  |
| P1 | GAATATATAAACAATGAAAGAAAGG | 319-332 | Cy3 |
| P2 | GCGGGGAGGACACACAGGTA | 355-372 | Cy3 |
| P3 | AATGAAAAGGCAGGTAAGTA | 399-418 | Cy3 |
| P4 | GAAGAAAAAAAGAATAAAAG | 473-496 | Cy5 |
| P5 | ACAAAAAGCAGGTATCCATG | 515-534 | Cy5 |
| P6 | GCAATAACTCACAAAACCAT | 741-761 | Cy3 |
| P7 | GGTTTTTTTTTTTTTTTTTT | 542-561 | Cy5 |
| P3' | AATGAAAAGGCAGGTAAGTA | 399-418 | Cy5 |
